# Hypervirulent pneumococcal serotype 1 harbours two pneumolysin variants with differential haemolytic activity

**DOI:** 10.1038/s41598-020-73454-w

**Published:** 2020-10-14

**Authors:** Stavros Panagiotou, Chrispin Chaguza, Reham Yahya, Teerawit Audshasai, Murielle Baltazar, Lorenzo Ressel, Shadia Khandaker, Mansoor Alsahag, Tim J. Mitchell, Marc Prudhomme, Aras Kadioglu, Marie Yang

**Affiliations:** 1 Department of Clinical Infection Microbiology and Immunology, Institute of Infection and Global Health, University of Liverpool, The Ronald Ross Building, 8 West Derby St, Liverpool, L69 7BE UK; 2 Wellcome Sanger Institute, Wellcome Genome Campus, Hinxton, Cambridgeshire CB10 1SA UK; 3 Darwin College, University of Cambridge, Silver Street, Cambridge, CB3 9EU UK; 4 Department of Veterinary Pathology and Public Health, Institute of Veterinary Science, University of Liverpool, Leahurst Campus, Neston, CH64 7TE UK; 5 Institute of Microbiology and Infection, College of Medical and Dental Sciences, University of Birmingham, Birmingham, B15 2TT UK; 6 Université Paul Sabatier, Centre National de la Recherche Scientifique, 118 Route de Narbonne, 31062 Toulouse Cedex 9, France; 7 Faculty of Applied Medical Sciences, Albaha University, Albaha, Kingdom of Saudi Arabia; 8 College of sciences and health professions, King Saud bin Abdulaziz University for Health Sciences, Riyadh, Saudi Arabia; 9 King Abdullah International Medical Research Center, Riyadh, Saudi Arabia

**Keywords:** Diseases, Infectious diseases, Bacterial infection

## Abstract

*Streptococcus pneumoniae* is a devastating global pathogen. Prevalent in sub-Saharan Africa, pneumococcal serotype 1 is atypical in that it is rarely found as a nasopharyngeal coloniser, yet is described as one of the most common causes of invasive pneumococcal disease. Clonal sequence type (ST)-306 and ST615 are representative of the two major serotype 1 lineages A and C, respectively. Here we investigated the virulence properties and haemolytic activities of these 2 clonal types using in vivo mouse models and in vitro assays. A lethal dose of ST615 administered intranasally to mice led to the rapid onset of disease symptoms and resulted in 90% mortality. In contrast, mice exposed to the same infection dose of ST306 or a pneumolysin (Ply)-deficient ST615 failed to develop any disease symptoms. Interestingly, the 2 strains did not differ in their ability to bind the immune complement or to undergo neutrophil-mediated phagocytosis. Upon comparative genomic analysis, we found higher within-ST sequence diversity in ST615 compared with ST306 and determined that ZmpA, ZmpD proteins, and IgA protease, were uniquely found in ST615. Using cell fractionation and cell contact-dependent assay, we made the unexpected finding that ST615 harbours the expression of two haemolytic variants of Ply: a cell-wall restricted fully haemolytic Ply, and a cytosolic pool of Ply void of any detectable haemolytic activity. This is the first time such a phenomenon has been described. We discuss the biological significance of our observation in relation to the aptitude of the pneumococcus for sustaining its human reservoir.

## Introduction

*Streptococcus pneumoniae* (or the pneumococcus) continues to thrive as one of the most lethal human pathogens globally. The pneumococcus is responsible for a broad spectrum of disorders ranging from mild infections, such as sinusitis and otitis media, to life-threatening diseases, e.g., community-acquired pneumonia, sepsis and meningitis. Described in 1918^1^, pneumococcal serotype 1 (S1) was the first serotype to be documented, and yet exhibits distinctive epidemiological, clinical and microbiological characteristics compared with the majority of the other 100 serotypes reported thereafter^2,3^. Over a century on, serotype 1 prevails as one of the most prevalent invasive serotypes, with a high incidence of disease reported globally, throughout Europe^4–9^, South America^10^, Asia^11,12^, Australia^13,14^ and particularly dominant in sub-Saharan Africa where it is responsible for devastating outbreaks of meningitis^15^. Most notably, pneumococcal serotype 1 is often referred to as a *high attack rate*^16^ pathogen as it is rarely detected in human nasopharyngeal swab specimens, yet it is associated with outbreaks and with the most lethal forms of invasive pneumococcal diseases (IPD).

In a former large-scale genomic analysis of serotype 1 clinical isolates, three distinct lineages were identified (lineages A, B and C) on the basis of their phylogenetic and geographical clustering. Lineage A includes ST306 and ST227, originating from Europe and North America respectively, while lineage B, mainly represented by the clonal complex ST217, was predominantly found in Africa and Israel. Lineage C isolates, mainly exemplified by ST615, originated from Chile^16^. In a more recent study, a 4th lineage (coined lineage D) was described for its strong association with clinical isolates clustered in Asia^16,17^. Intriguingly, lineages A and C were reported to exhibit residual or no haemolytic activity, attributed to the expression of a non-haemolytic variant of pneumolysin (Ply) or to its null expression^8,18,19^. Given the critical role attributed to Ply and its haemolytic activity in enhancing pathogenicity, we raised the question as to whether common mechanisms and/or unknown virulence factor(s) may have compensated for the loss of haemolytic activity, which allowed both lineages A and C to thrive as invasive isolates^20^. In the present study, we sought to understand the virulence properties of these two lineages, using ST306 and ST615 as their respective representatives.

The peculiarity of the ST306 clone lies in its expression of a non-cytolytic Ply variant—which has essentially conserved its capacity to bind to cholesterol present in cell membranes, but lost its pore-forming functionality^8^. Clinically, ST306 is typically associated with non-lethal pneumonia with or without empyema and is documented to be preferentially linked with recurrent paediatric infections^21^. Previous studies have proposed that expression of a non-haemolytic variant of Ply conferred an evolutionary advantage to the pneumococcus from the perspective of immune evasion strategies^22,23^ e.g., lack of NLPR3 inflammasome activation with significantly reduced production of IL-1β^24^. Considerably scarcer information is available on the ST615 clone and its Ply gene variant. First isolated in New York in 1948^10,25^, ST615 was much later documented as the causative agent of a pneumococcal serotype 1 IPD outbreak that occurred in Chile^16^. Besides ST615, one other lineage C isolate (i.e., ST3018, Ply variant 4496) was documented, which produced no or very low specific haemolytic activity, i.e., 1.6% that of D39-derived Ply^22^.

Of the many virulence factors associated with the pneumococcus, Ply is central to its pathogenesis. Its cytolytic and pro-inflammatory activities, in particular, are often described as the primary mechanisms contributing to colonisation of the airways^26,27^, as well as invasion and extra-pulmonary dissemination^28,29^. We previously reported its TLR4-independent activation of the NLRP3 inflammasome^24^, its role in the establishment and maintenance of nasopharyngeal carriage^30^ and its binding to mannose receptor C type 1^31^. Previous studies also documented the ability of Ply domain 4 to promote activation of both the classical and lectin pathways of complement activation^32^, host-to-host transmission^33^, necroptosis^34^, platelet activation^35^, and biofilm formation^36^-independently of its haemolytic activity^37^. Given the pivotal role of Ply in the pathogenesis of IPD, its conserved expression, and its contribution to organ damage^38^ and dysfunction, a myriad of studies have explored its use as a therapeutic target and/or a universal vaccine candidate^39,40^.

An interesting feature of Ply, documented in studies by Camilli and Price^41,42^ was that in certain serotypes including 3, 4 (TIGR4) and 8, it could localise to the pneumococcal cell wall. Despite these findings, no subsequent studies were carried out to systemically test for the presence of Ply in the cell wall of other clinically relevant pneumococcal serotypes. Instead, subsequent reports continued in measuring haemolytic activity or expression of Ply as an actively secreted cytosolic protein or as a toxin released upon bacterial lysis. Here, we made the unexpected finding that ST615 produces two Ply variants; a haemolytically active cell wall-associated version and a non-haemolytic cytosolic variant. Our study reignites the paradigm that selective host or environmental pressures may exist which drive the pneumococcus to adapt and develop concealment or compartmentalisation strategies to guard its key virulence factors and sustain its global pool of circulating pneumococci.

## Results

### ST615 is hypervirulent compared with ST306 in invasive pneumonia mouse model

**Figure 1:**
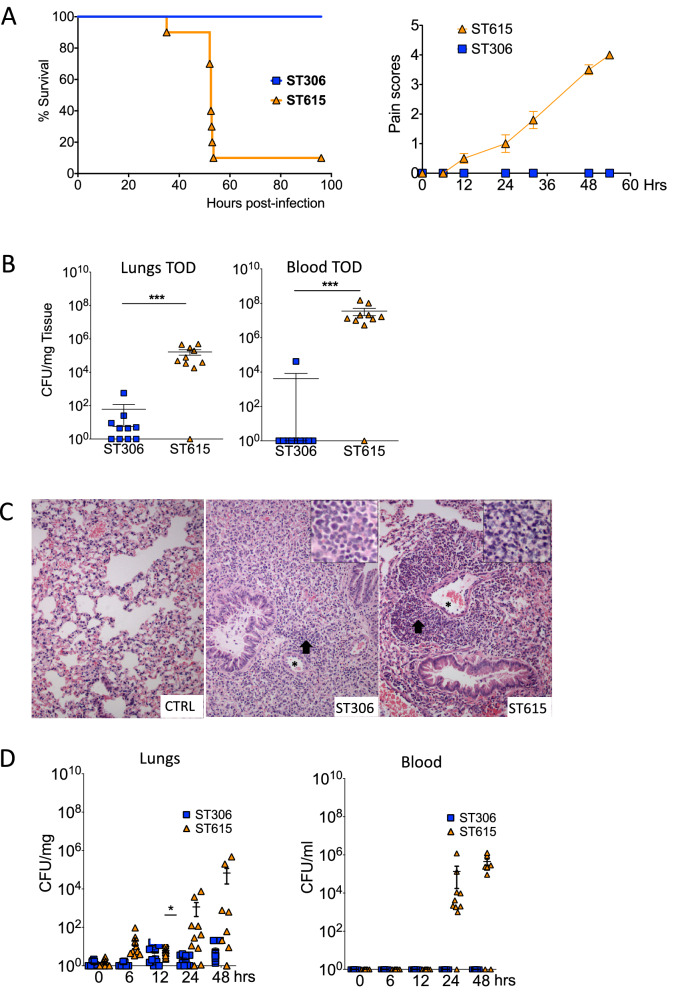
ST615 is more virulent than ST306 pneumococcal clone. (**A**) Comparative study using a mouse model of invasive pneumonia. Kaplan–Meier survival chart monitoring the number of mice succumbing to disease over 96 h. At the experimental endpoint, all mice (100%) challenged with ST306 survived as opposed to 10% survival only in mice challenged with ST615 (n = 10 mice per group, Log-rank test, ***p < 0.0001). Upon challenge with pneumococci, mice were monitored for the development of visual signs of disease (n = 10 mice per group; mean ± SEM). Pain scores were assigned as follows 1: hunched, 2: starry/piloerecti, 3: lethargic, 4: moribund, 5: dead. (**B**) Pneumococcal viable counts (CFU/ml) in lung tissues and peripheral blood upon intranasal infection of mice with ST306 compared with ST615 pneumococci at time of death (TOD). Data are represented as mean ± SEM (n = 10 mice per group). Each mouse is represented by a symbol. Statistical significance (Student t-test, ***p < 0.001). (**C**) Histological analysis of lung tissues collected from ST306 versus ST615-challenged mice using an invasive pneumonia infection model compared with naïve control (CTRL). Asterisks indicate vessels while arrows represent inflammatory cell infiltration. Inset: magnification of inflammation area. Original magnification × 20 (inset 40 ×); scale bar 50 microns (inset: 20 microns). (**D**) Kinetics of infection as monitored by pneumococcal viable counts. Mice were challenged intranasally with 10 μl of sterile PBS containing 1 × 10^6^ CFU/mouse of ST306 or ST615 pneumococci. Mice were culled at the indicated time points and viable counts were determined by the Miles and Misra technique in homogenised lung tissue and blood. Data are represented as mean ± SEM, n = 10 mice per group per time point.

To compare the invasiveness of ST615 and ST306 isolates in vivo, groups of mice were intranasally challenged with an inoculum containing equal viable counts of each respective strain. All mice infected with ST306 showed no signs of disease and all survived the challenge, while 90% of ST615-challenged mice succumbed to the infection (Fig. 1A, left panel). In line with this, ST615-challenged mice presented increased signs of disease between 6 and 12 h post-infection (Fig. 1A, right panel). A significant difference (*p* < 0.05) was determined in the number of viable colonies found in the lungs and blood of mice challenged with ST615 versus ST306 (Fig. 1B). No bacteria were detected in blood of mice challenged with ST306—except for one mouse presenting viable counts in the order of 10^3^ CFU/ml, while all ST615-infected animals reached levels of the order of 10^7^ CFU/ml. ST306 pneumococci were found in lung tissues at a density 3-log lower than that of ST615-challenged mice (*p* < 0.01). Our findings are consistent with previous studies reporting the weak virulence properties of ST306^8^, as opposed to the hypervirulence of ST615^43^.

Upon histological analysis, we showed that lung tissue collected from naive mice did not exhibit any histopathological signs, while lungs from ST306- and ST615-infected mice showed predominantly perivascular and to lesser extent peribronchial aggregates of inflammatory cells (Fig. 1C). Lung tissues in the ST306 group exhibited a diffuse infiltration of non-degenerated neutrophils and only a small number of lymphocytes. On the other hand, the lung tissues of ST615-infected animals presented a higher density of inflammatory cells, with necrotic neutrophils. These observations corroborate with the histological features previously described in human patients^21,44^.

**Figure 2:**
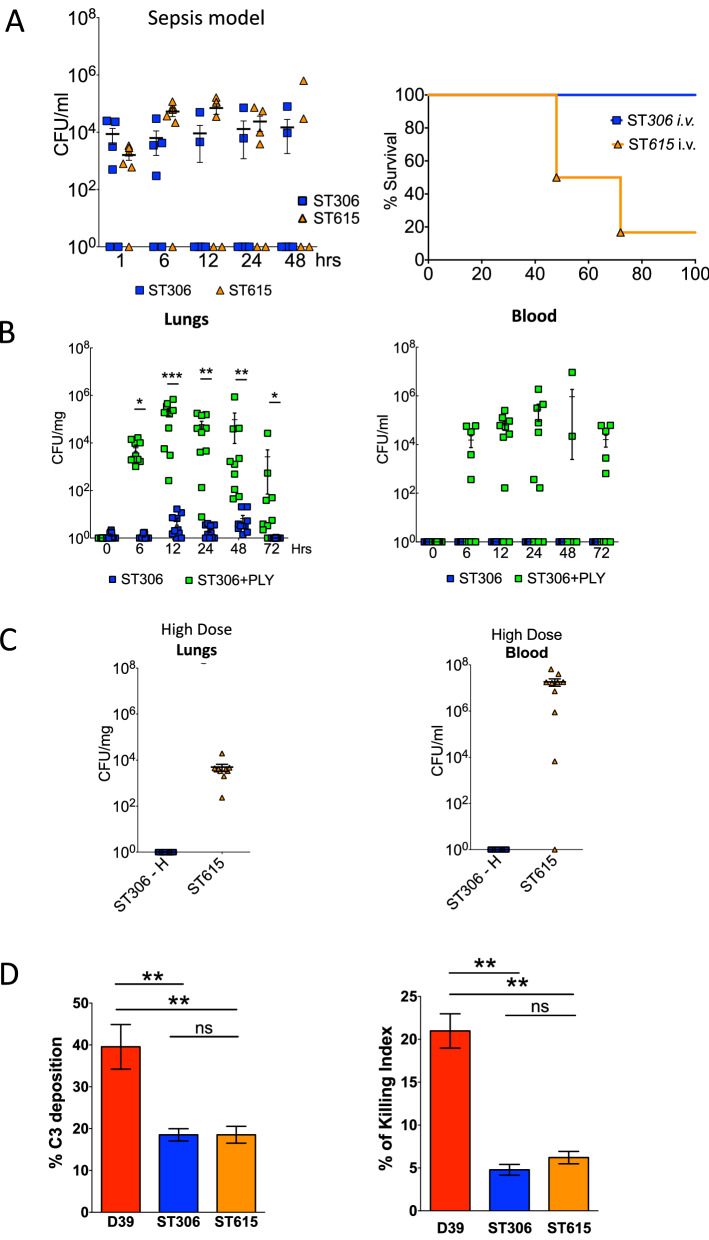
Haemolytically potent Ply promotes invasion into the lung and blood of ST306-infected mice (**A**) Mice were challenged intravenously with 100 μl of sterile PBS containing 1 × 10^6^ CFU/mouse of ST306 or ST615 pneumococci. Whole blood was collected via tail bleed at the indicated time points and viable counts were determined by Miles and Misra (left panel). Kaplan–Meier survival chart (right panel) monitoring the number of mice succumbing to disease over 96 h in the same mouse sepsis model. (**B**) Serotype 1 ST306 was mixed with pneumolysin allele 1 (100 ng/mouse, 100,000HU/mg) and administered intranasally in a 50 µl volume/mouse. Viable counts were monitored in lung tissues and in blood at 0, 6, 12, 24, 48 and 72 h post-pneumococcal administration. Data are represented as mean, n = 10 mice per group ± SEM (*p < 0.05, **p < 0.01, ***p < 0.001). (**C**) Mice were challenged intranasally with 50 μl of sterile PBS containing either 1 × 10^7^ CFU of ST306 (coined ST306-H) or 1 × 10^6^ CFU of ST615 pneumococci. Viable counts in the lung tissues and blood were determined at the experimental endpoint. Data are represented as mean ± SEM, n = 10 mice per group. (**D**) Plots showing percentage of in vitro C3 complement deposition (left panel) and HL-60 killing index (right panel) in pneumococcal strain D39 versus serotype 1 clones ST306 and ST615. One-way ANOVA with Tukey’s post hoc analysis, **p < 0.01; ns: non-significant.

We then sought to determine whether and how the kinetics of infection differed between the two strains within the first 48 h post-infection. Using our standardised model of invasive pneumonia, mice challenged with ST615 presented 2 to 3-log higher bacterial loads in their lung tissues at 6, 24 and 48 h post-infection compared with ST306, with mounting bacterial densities over time (Fig. 1D). Similarly, ST615 bacteria were detected in the blood at 24 and 48 h post-infection, while none of the ST306-challenged mice presented any viable colonies. In view of reports suggesting that expression of Ply with low haemolytic activity could provide an early growth advantage when administered directly in blood^19^, we examined the in vivo proliferation of ST306 and ST615 in blood using a mouse model of sepsis (Fig. 2A). In our hands, no significant difference was found between the respective viable counts of the two clones over the 48 h-monitoring period. A striking distinction, however, was that 80% of mice infected with ST615 pneumococci succumbed to the infection (Fig. 2A), while all ST306-intravenously challenged mice survived the infection and did not exhibit any visual signs of disease. Our results showed that, despite both mouse groups showing similar bacterial densities in blood i.e., 10^4^–10^5^ CFU/ml, the inherent hypervirulence of ST615 led to a lethal outcome.

### ST306 becomes invasive but remains avirulent upon co-administration of haemolytically active pneumolysin in vivo

To further examine the importance of Ply and its haemolytic activity in pneumococcal pathogenicity, we conducted a supplementation experiment whereby ST306 pneumococci were co-administered with haemolytically active endotoxin-free recombinant Ply in vivo. We observed a significant increase in ST306 viable counts in lung tissue from 6 h onwards. This was accompanied by the translocation of ST306 from lung tissue into blood—peaking at 24 h post-challenge (Fig. 2B). Interestingly, despite the presence of significant bacterial loads in lungs and blood, the mice failed to show any visual signs of disease. Pneumococci expressing non-haemolytic Ply, such as ST306, are known to be associated with lower case-fatality rates^45^ and known to be less efficiently recognised by the innate immune system^22,46^. Altogether, these properties might have contributed to the capacity of ST306 to invade sterile compartments without triggering any disease symptoms.

To determine whether the hypovirulence of ST306 compared with ST615 may be compensated, a tenfold higher (10^7^ CFU/mouse) challenge dose of ST306 was administered to mice—in comparison to our standardised 10^6^ CFU/mouse (used in Fig. 1D). The results showed either a residual presence or total absence of any detectable ST306 pneumococci in lungs and blood, respectively (Fig. 2C).

In view of the divergent virulence properties observed between ST306 and ST615 in vivo, we sought to detemine whether these dissimilarities may be mirrored in their properties to evade the host immune system. To this end, opsonophagocytosis killing (OPKs) and C3-complement binding assays were carried out (Fig. 2D). Our results indicated that ST615 and ST306 did not exhibit any significant differences in their ability to resist either complement deposition or phagocytic uptake.

### Comparative genomic analysis of ST306 and ST615 clonal lineages and *Ply* phylogeny

**Figure 3:**
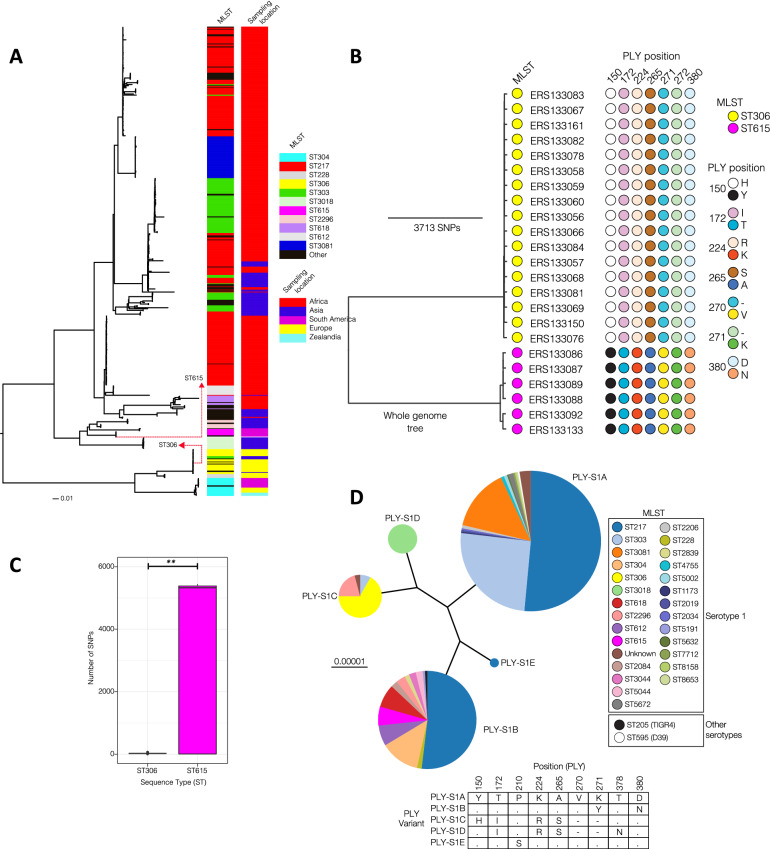
Comparative genomic analysis and mapping of ST306 and ST615 clonal complexes across 5 continents. (**A**) Maximum likelihood phylogenetic trees showing the geographical clustering and relationship between ST306 and ST615. Sequence cluster (SC) represents a collection of isolates from the same clade, but which may be of different serotypes and STs. The SCs were identified using the sequence clustering software BAPS. (**B**) Pneumolysin sequence alignments. Amino acid sequence alignments for ST306 and ST615 pneumolysin compared with D39 pneumolysin sequence. Left panel: nucleotides, Right panel: amino acids. (**C**) Within ST nucleotide sequence diversity estimated by pairwise comparison of the genomes from each ST to determine number of single nucleotide polymorphisms (SNP) between pairs of isolates. The 95% confidence intervals for the SNP diversity plots are: ST615 (mean: 2883.60, lower: 1.00 and upper: 5445.55) and ST306 (mean: 26.96, lower: 7.38 and upper: 61.00, Student t-test, *p* = 0.0014) (**D**) Phylogeny/dendrogram of all serotype 1 Ply alleles (all STs with D39 and TIGR4). A total of 488 clinical isolates were used for this analysis. For comparisons, Ply alleles of TIGR4 (serotype 4) and D39 (serotype 2) laboratory strains are also included. Different colours show STs associated with each serotype 1 Ply allele. The specific amino acids that distinguish the Ply alleles are shown in the table below the Ply phylogeny. The different serotype 1 Ply alleles were arbitrarily labelled as PLY-S1x where the suffix ‘x’ corresponds to letters A to E for the five alleles identified.

Global analysis of pneumococcal serotype 1 strains showed that the majority of the clones exhibited phylogeographical structure. When placed in a global context, ST615 strains appear to be localized in South America while ST306 strains show a relatively higher geographical spread than ST615 although they were primarily detected in Europe (Fig. 3A). The two clones appear to be highly divergent and do not cluster together in a single clade, suggesting that the two clones may exhibit notable phenotypic differences owing to their genetic differences. Using a genomic database of 23 isolates originating from 3 different continents, we confirmed previous findings that all ST306 strains expressed the Ply allelic variant 5 in a very conserved manner. This allelic variant differs from D39 allele 1 by four amino acid substitutions (Y150H, T172I, K224R, and A265S) and two amino acid deletions (V270- and K271-) (Fig. 3B). The non-haemolytic allelic variant 5 Ply is also expressed in other MLST serotype 1 e.g., ST228, ST617, and serotype 8 e.g., ST53, ST578, ST835, ST1110, ST1722^18^. In line with previous studies^18^, we determined that ST615 Ply differs from D39 allele 1 by only one amino acid substitution at position 380 (D380N), similarly to the TIGR4-associated allele 2 (Supplemental Table 2). At gene level, ST306 and ST615 strains share 71% (1768/2482) common core genes while 39% (714/2482) constitutes the accessory (flexible) genes not common to both clones. The differences between the two clones may be due to variations in alleles of important virulence factors (e.g. pneumolysin) and presence of clone-specific accessory genes. There is higher within-ST sequence diversity in the ST615 (mean: 2884, range: 1 to 5488 SNPs) than ST306 (mean: 30, range: 5 to 66 SNPs) (*p* = 0.0014, Student t-test) (Fig. 3C). While ST615 clone is less common compared with ST306, it is more genetically diverse possibly reflecting higher recombination or an older most recent common ancestor, which has undergone more diversification over time.

Phylogenetic trees were constructed based on the nucleotide sequences of the entire *ply* gene across a total of 488 pneumococcal serotype 1 clinical isolates (Fig. 3D), in comparison to D39 and TIGR4. Each allelic variant was assigned to one of 5 allele groups (A, B, C, D or E) according to the main branch from which it was derived. Clades A and B were associated with the most diverse sequence type distribution. As expected, Ply allele 2 clustered within group C whereas allele 5 was placed in the furthest diverged group A.

To further comprehend the heterogeneity between ST306 and ST615, we exploited genomic data encompassing a larger collection of pneumococcal isolates originating from 5 distinct continents and found approximately 15,799 (range: 10,272 to 16,635) single nucleotide polymorphisms in core genes between ST306 and ST615, and also differences in 588 accessory genes: 192 of which were unique to ST306 and 198 unique to ST615. To investigate differences in the accessory elements, we adopted a straightforward absence/presence analysis of individual genes. Accessory regions present in all ST306 isolates but absent in ST615 isolates included among others, regions encoding bacteriocin peptides, a number of ABC transporter/permease ATP-binding proteins, a DNA integrase and a fucose operon protein^47^, a number of DNA methylases and methyltransferases, and a helicase. Most interestingly, the presence of ZmpA and ZmpD proteins, and IgA protease, were uniquely found in ST615, offering altogether another basis for its significantly more pronounced virulence compared with ST306. Subsequent pan-genome analysis using the Scoary software^48^ detected 396 accessory genes that were specific to a single clone (Supplemental Table 3). These genes exhibited different functions including nutrient transport, methylation, immunity (bacteriocins), two-component systems, transcription regulation, drug resistance and competence. By far the most common clone-specific genes appeared to be associated with mobile genetic elements such as integrases, transposons, insertion sequences and phages. It is important to point out that, while sequence-types defined by MLST do not necessarily define a clone, here clonality and serotyping were inferred from whole genome sequencing data. In the future, as our database of pneumococcal genomes continues to grow, we hope to extend the present findings to a larger and globally relevant collection of isolates.

### ST615 harbours the expression of a mixed pool of haemolytic and non-haemolytic pneumolysin

**Figure 4:**
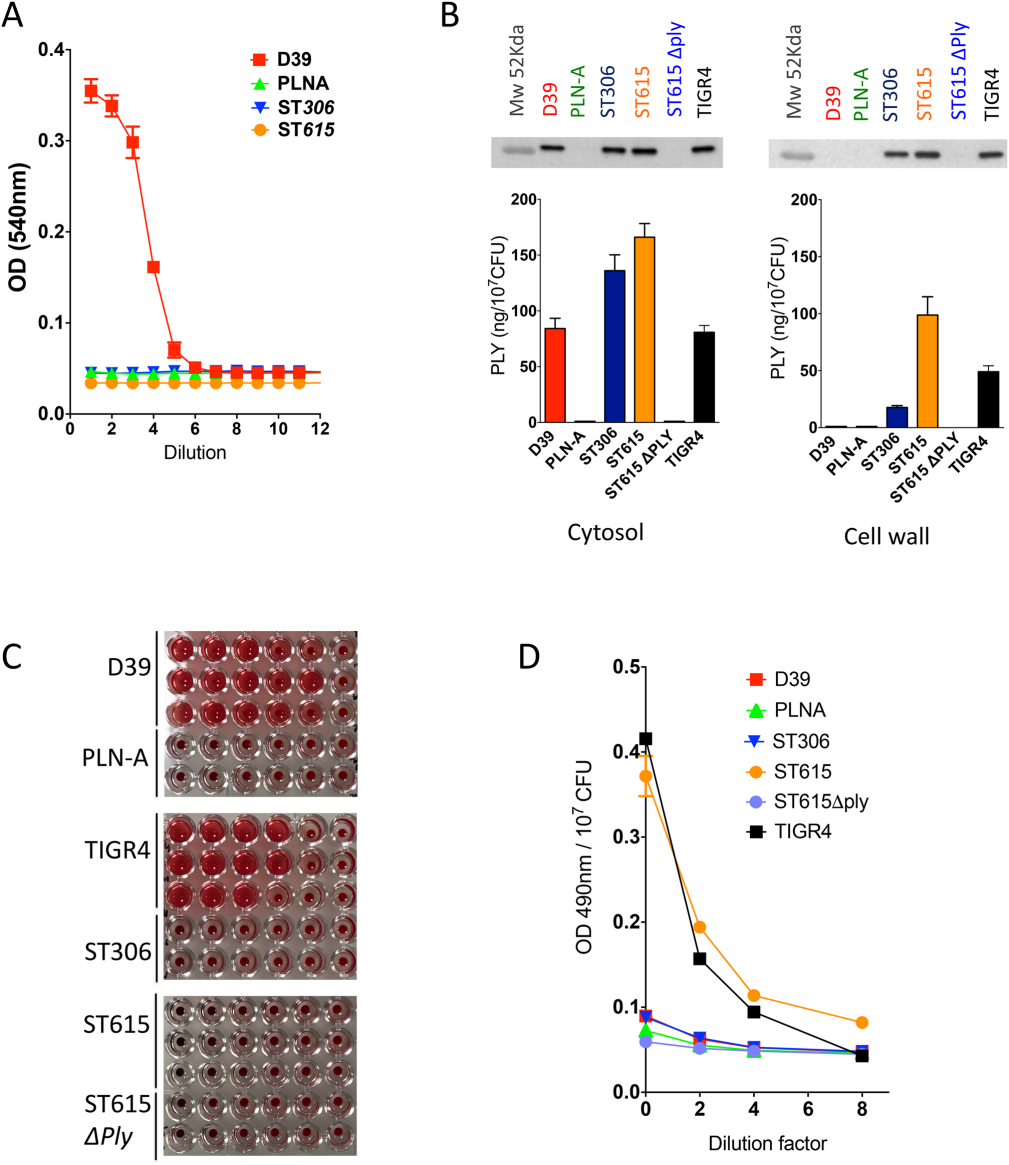
Pneumococcal ST615 present 2 Ply pools: cell-wall associated versus cytosolic variant. (**A**) Determination of haemolytic activity using the standardised sodium deoxycholate-based extraction assay. Serial dilutions were carried out from fresh cultures of bacterial stock at 10^8^ CFU/ml and subsequently incubated with a 4% sheep red blood cell solution. The data are representative of 3 independent experiments (n = 3 replicates, mean ± SEM) (**B**) Detection and quantification of pneumolysin in the cytosol (left) and cell wall (right) fractions of the indicated pneumococcal strains via Western blot and ELISA analysis using an anti-Ply mouse monoclonal antibody. (**C**) Determination of the haemolytic activity of the Ply-containing cytosolic fractions (**D**) Contact-dependent haemolysis was tested in ST615, ST306, TIGR-4, D39 and *ply*-knockout ST615. PLN-A was used as a negative control.

Using a previously standardised haemolytic assay^18^, both ST306 versus ST615 were found to be non-haemolytic i.e. unable to lyse red blood cells (Fig. 4A), in sharp contrast with D39. The Ply deficient isogenic mutant of D39 (PLN-A) was used as a control and showed no detectable haemolytic activity^49^. Using a previously reported fractionation protocol, pneumococcal cell wall proteins were separated from the cytosolic fraction and subjected to quantification of pneumolysin by ELISA and Western blot analysis. The presence of Ply was found in the cell wall fraction of both ST615 and ST306 clones, where ST615 had a twofold and fivefold higher concentrations compared with TIGR4 and ST306, respectively (Fig. 4B). Ply was also found to be expressed in the cytosol of ST306 and ST615, while it was exclusive to the cytosolic fraction of D39. Because the concentrations of Ply measured in the cytosolic fraction of ST615, ST306 and TIGR4 were consistently lower than those found in their respective cell wall fractions (Fig. 4B), we rule out a non-specific leakage from cytosolic to cell wall fraction.

As expected, the cytosolic fractions of D39 and TIGR4 both presented haemolytic activity, however, we made the unanticipated finding that the cytosolic fraction of ST615 was void of any haemolytic activity (Fig. 4C), despite the presence of Ply confirmed by ELISA and Western blot analysis. A contact-dependent assay was then carried out to interrogate the existence of a cell-wall associated lytic activity. The results showed significant red blood cell lysis activity in ST615 and also TIGR4 (Fig. 4D), in contrast with the absence of any contact-dependent haemolytic activity in ST306, D39 or PLN-A.

**Figure 5:**
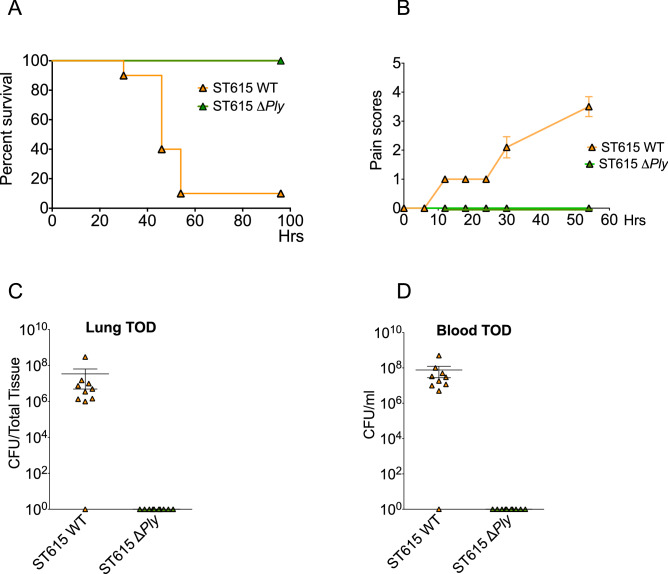
Pneumolysin is indispensable to the invasive properties of pneumococcal ST615. (**A**) Upon challenge with ST615 wild-type (WT) or ST615Δ*ply* pneumococci, mice were monitored for the development of visual signs of disease (n = 10 mice per group; mean ± SEM). Pain scores were assigned as follows 1: hunched, 2: starry/piloerecti, 3: lethargic, 4: moribund, 5: dead. (**B**) Kaplan–Meier survival chart monitoring the number of mice succumbing to disease over 3 days upon challenge with ST615 WT versus ST615Δ*ply*. (**C**,**D**) Pneumococcal viable counts in lung tissues (**C**, CFU/tissue) or and peripheral blood (**D**, CFU/ml) upon intranasal infection with ST615Δ*Ply* compared with ST615 WT pneumococci at time of death (TOD). Data are represented as mean ± SEM (n = 10 mice per group). Each mouse is represented by a symbol.

To further assess the role of Ply in the pathogenicity of serotype 1/ST615, we genetically knocked-out its expression of *ply* and compared the virulence of the deletion mutant to its wild-type counterpart using a mouse model of invasive pneumonia. Mice challenged with ST615Δ*ply* all survived the pneumococcal challenge, while only 10% of mice administered with the wild-type parent ST615 strain survived (Fig. 5A). Mice infected with ST615Δp*ly* exhibited no visual signs of disease (Fig. 5B) and had no detectable CFU in neither lung nor blood at the experimental endpoint (Fig. 5C,D). The absence of cell-wall associated haemolytic activity in the ST615Δ*ply* mutant strain was confirmed in our contact-dependent assay.

## Discussion

Pneumococcal serotype 1 is one of the most invasive and deadly pneumococcal strains globally, especially in resource-poor settings. However, our current understanding of the mechanisms underlying its pathogenicity is limited. The discovery of serotype 1 clones such as ST306 and ST615, has contributed further to the complexity of serotype 1 pathogenesis. These two clones belong to two distinct geographical and phylogenetic lineages, yet they share the property of presenting non-^8^ or residual haemolytic^19^ activity. Both clones are associated with IPD outbreaks and present the intriguing distinction that ST306 is mildly virulent and rarely lethal, yet causes pneumonia and empyema^44^, while ST615 exhibits virulence properties similar to fully haemolytic serotype 1 pneumococci^19^. Here, we report the unexpected finding that ST615 harbours the expression of two Ply variants i.e., a cytosolic non-haemolytic Ply and a fully haemolytic cell-wall associated Ply variant.

We confirm the role of Ply in the hypervirulence of ST615 by demonstrating that survival rates of animals exposed to ST615Δ*ply* increased from 10 to 100% in an invasive pneumonia model. Our results corroborate with those of Camilli and Price (2009) who previously showed that Ply could localise to the cell wall of select serotypes. Notably, ST615 showed a fivefold higher expression of Ply in its cell-wall fraction compared with ST306, which may have contributed to its propensity to transit faster from lungs to blood. In our hands, Ply protein expression was absent in the cell wall fraction of D39, while it was detected in both the cell wall and cytosolic fractions of both ST615 and ST306. We made the unanticipated finding that cytosolic Ply of ST615 was void of any detectable cytolytic activity, as opposed to its cell wall-associated counterpart, which exhibited full haemolytic activity.

On the basis that ST615 present only one copy of the *ply* gene, we rule out that genomic variations may provide an explanation for our observation. Instead we favour the hypothesis that the non-haemolytic cytosolic Ply variant may be the result of protein–protein interactions whereby an unknown accessory protein, possibly specific to ST615 and related clones, may actively bind to the cytosolic Ply pool thus preventing its oligomerisation and pore-forming functionality e.g., in a manner similar to toxin-antitoxin systems previously described in the pneumococcus^50^. Another scenario may be that the cytosolic pool of Ply may simply represent a reservoir of monomeric Ply precursors in transit to the cell wall compartment, where it will embed and oligomerise.

While the non-haemolytic ST306 clone was described over a decade ago^8^, the evolutionary significance of non- or weakly haemolytic Ply variants strains remain elusive. Given the documented expansion of ST306-related IPD in Europe, and its high incidence rates in Africa and South America, we are in favour of the theory that a deficiency in the haemolytic activity of Ply represents an evolutionary adaptation beneficial to the pneumococcus, rather than a disadvantage. Previous authors^8,18,51^ have surmised that such clones may represent a self-sustainable reservoir of quiescent strains capable of altering their genomic composition in response to environmental selective pressures such as climate and/or human-led interventions e.g., immunisation.

While previous epidemiological studies carried out in North America and in Europe reported that ST306 might have emerged concomitantly or as a result of the introduction of pneumococcal conjugate vaccines^44,52^, other authors have clearly established that both ST306 and ST615 serotype 1 clones existed prior to the introduction of PCV-7^53^. Given the phylogenetic distance between ST615 and ST306, we do not rule out that differences in their virulence also results from clone-related variations between their genomes. Indeed, our comparative genomics analysis showed that a number of accessory regions were uniquely found in ST615 isolates, including among others ZmpA/ZmpD proteins, and IgA protease. Whether these accessory proteins contribute to the mixed Ply phenotype of ST615 remain to be established. We compared the *ply* alleles found in ST615 and ST306 with the *ply* alleles found in other serotype 1 strains: the ST615 *ply* was similar to one of the alleles for the virulent ST217 clone allele while the ST306 *ply* allele was distinct. These *ply* polymorphisms may have contributed to differences in virulence between ST615 and ST306 strains. The exclusive presence of immunoglobulin proteases—such as ZmpA (also known as IgA protease) and ZmpD proteins (cleaves the human immunoglobulin A (IgA) to evade complement-mediated or mucociliary clearance^54^)—which were uniquely found in ST615, offer another basis for its significantly more pronounced virulence compared with ST306.

The *ply* allelic variant 2 was described in other serotypes i.e. 3, 35F, 4,14, 34, 19A, 11A, 16F, 17F and 38, spanning at least 27 different STs^51^ of both carriage and invasive origins. In the absence of a clear association between *ply* allelic variants, such as the allele 2, and pneumococcal serotype or invasive potential^18,51^, one could argue that genetic background, more than *ply* variant, is the main driver of diversity in pneumococcal virulence. Here, we argue that the higher protein expression levels of Ply combined with its cell wall compartmentalisation significantly contribute towards the hypervirulence of ST615.

A closer examination at the flanking genomic regions of ST615 *ply*, confirmed the lack of a leader signal peptide^41,42^ as well as that of any classic membrane-anchoring motifs^55^. This is highly reminiscent of the characteristic features of non-classical surface proteins expressed by pneumococci and many other Gram-positive bacteria. These proteins are also commonly known as ‘*moonlighting*’ proteins^56,57^ because they frequently present more than one function or activity depending on their anatomical locations i.e., cytosol versus cell wall. PavA, eno and GAPDH are examples of such moonlighting proteins in *S. pneumoniae*^58^. Here we propose that Ply may be one such non-classical cell wall-associated protein. While knowledge on the export or anchoring mechanisms of these non-classical surface-associated proteins remains obscure, we previously reported that their transport may be dependent on the accessory SecY2A2 system^59^. Given that ST615 and TIGR4 express the same *ply* allele, it is likely that the two strains share a Ply transport mechanism to the cell wall that is distinct from or absent in strains such as D39.

Our observations leave open the question of the biological significance of a cell wall-associated toxin versus a cytosolic/secretory toxin. Intuitively, a cell wall-shielded toxin may be less prone to enzymatic degradation and/or immune recognition by the host, hence promoting survival of the bacterium in vivo. Here, we showed that ST615 and ST306 did not differ in their propensity to bind to immune complement or to be phagocytosed by human neutrophils. Hence, an alternative scenario may be that cell-wall associated Ply acts as a ligand for host cell surface receptors in vivo, as recently shown for Ply binding to macrophage and dendritic cell mannose receptor^31^, to mediate and or interfere with a range of host immune signalling pathways. The results from our contact-dependent haemolytic assay also raises the possibility that a cell-wall associated Ply may contribute to functions such as contact-dependent antagonism^60^, whereby pneumococci may compete with other bacterial species for nutrients and space within a tissue niche, to promote colonisation and pathogenesis.

Similarly, we cannot rule out that a specific trigger may exist in vivo that stimulates Ply release from the cell wall of ST615. It has been documented indeed that, during the invasion process, when the pneumococcus traverses tissue niches with varying temperatures, nutrients and oxygen levels, the bacterium is capable of *smart* regulating its growth pattern and/or *ply* expression^61–63^ in response to rapid changes in its external environment. In view of a recent report that *S. pneumoniae* is capable of replicating inside splenic macrophages^64^ and that it can create pores in the mitochondria of neurons^65^, we propose that a cell wall-associated Ply provide an advantage for the pneumococcus to survive as a pathogen during its intracellular lifestyle. For instance, other Gram-positive microorganisms such as *Listeria monocytogenes* make use of its toxin to escape from phagolysosome^66^, while other streptococcal species, such as *Streptococcus agalactiae*, share the property of presenting a cell-wall associated haemolysin^67^, which plays a critical role in its pathogenicity.

Ply is classically known for its affiliation to the protein family of cholesterol-dependent cytolysins (CDC) and their characteristic α-haemolytic activity. Our findings suggest that the ST615 cell-wall associated Ply variant is more effective in its pore-forming oligomerisation capacity than its cytosolic counterpart. In the last decade, it has become increasingly documented that CDC proteins play much more sophisticated roles than that of simple pore-forming lytic toxins. This is reflected in their ability to activate complement, to bind to nonsterol receptor, to function as a protein translocation channel or to induce DNA damage^68–72^. ST615 pneumococci may have undergone unique evolutionary changes resulting in the production of two pools of Ply with distinctive functions so that upon direct contact with host cells, its cell-wall associated Ply readily form pores or readily induce cell cycle arrest while, upon release, its cytosolic non-haemolytic Ply pool readily activate complement, bind to immune cell receptors or triggers other yet unknown mechanisms. This scenario is consistent, for instance, with our earlier reports that the pore-forming and complement activation activities of Ply contribute distinctively to the pathogenicity of *S. pneumoniae*^73^.

In summary, our findings reaffirm the role of Ply as a critical pneumococcal virulence factor and highlight a previously unreported phenomenon of pneumococci harbouring both lytic and non-lytic Ply variants. Our study also reinforces the paradigm that the pneumococcus is an incredibly versatile microorganism capable of devising strategies to adapt its armoury of virulence factors and persist as an important human pathogen, whether intracellularly or extracellularly. Understanding of the genomic variation that led to the emergence of unique clones such as ST615, and dissection of the clone-specific mechanisms controlling the expression and compartmentalisation of key virulence factors such as Ply, will help design more efficient strategies towards combatting invasive pneumococcal disease.

## Material and methods

### Pneumococcal strains

Pneumococcal identification was confirmed by optochin sensitivity test and serotypes were determined using the standard Quellung reaction with pneumococcal capsule-specific antisera (Statens Serum Institute, Copenhagen, Denmark). All isolates were stored in the lab at − 80 °C on PROTECT cryobeads (Pro-Diagnotics Lab, Inc.) and subcultured on 5% horse blood agar plates incubated at 37 °C under anaerobic conditions for 18 h. Multilocus sequence typing (MLST) was carried out on all isolates. The two clinical isolates i.e., ST306 and ST615, originate from archives collected between 2001 and 2008 in the context of surveillance programmes established in France (Prof Francois Trottein, Pasteur Institute, Lille, France) and Uruguay (Chabalgoity JA, Universidad de la República, Montevideo, Uruguay), respectively. Both isolates were collected from IPD patient blood cultures and selected as representative isolates of pneumococcal serotype 1 lineages A and C. Pneumococcal strains D39 (ST595, NTCT 7466) and TIGR4 (ST205, NTCT 7465) were used for comparative analysis^74,75^.

### Construction of pneumococcal serotype 1/ST615Δ*Ply* mutant

Generation of competent *S. pneumoniae* cells and subsequent transformation were performed using the complete transformation medium (C + Y media, pH = 6.8) method^76^. Mutants were generated using the primers detailed in Supplemental Table 1. *Ply*-knockout ST615 was constructed by replacing the *ply* open reading frame (ORF) with the *aphA3* cassette (conferring kanamycin resistance) and subsequent selection of kanamycin-resistant recombinants, ST615*ply::aphA3*, on blood agar base medium supplemented with kanamycin. The desired recombination was verified by sequencing.

### Ethics and mouse challenge experiments

All animal experiments were conducted in accordance with the Animals (Scientific Procedures) Act 1986 and Amendment Regulations 2012 (ASPA 2012), and the care and maintenance guidelines of the Universities of Liverpool and Glasgow. All animal protocols were approved by the Local Animal Welfare and Ethics Committees under the UK Home Office Project Licence PB6DE83DA. In line with the 3Rs principle, the number of animals was kept to a minimum. Age-matched (6–8 weeks) outbred MF1 female mice (Charles River, Margate, U.K.) were used in the pneumonia and sepsis models. The pneumococcal isolates (ST306 or ST615 clones) were streaked onto 5% blood agar plates. Single colonies were grown overnight in BHI, and then subcultured in BHI + 20% serum for storage at − 80 °C. Inocula were administered to mice in sterile PBS vehicle solution at varying densities: in the pneumonia model, mice were anesthetized and infected intranasally with 10^6^ CFU/50 μl/mouse, while in the sepsis model, mice received an intravenous administration of 10^6^ CFU/100 μl/mouse. In this latter model, tail bleeds were performed at 6, 12 and 24 h post-administration to monitor the kinetic of infection and determine viable bacterial counts. The total duration of the sepsis experiment was 48 h. To assess the role of haemolytically active pneumolysin, groups of mice were intranasally co-administered 10 ng/g body weight of recombinant Ply (100,000 HU/mg) in 10^6^ CFU/50 μl sterile PBS. The animals were monitored for a total of 10 days. Blood, nasopharynx and lung tissues were homogenized and plated onto blood agar base (BAB) plates for CFU determination using the Miles and Misra method^77^. These BAB plates were supplemented with horse blood (5% final concentration) and 5 µg/ml of gentamycin. Gentamicin is a selective agent inhibitory to most *Enterobacteriaceae*, *Neisseria, and Staphylococcus* spp*.* After incubation, plates were further examined for colony morphology and haemolytic zone characteristic of pneumococci, and in some cases, optochin sensitivity was also confirmed.

### Determination of cytosolic haemolytic activity

Determination of the cytosolic haemolytic activity of pneumococcal serotype 1 ST306 and ST615, as well as serotype 2 D39 (ST128) and serotype 4 TIGR4 (ST205) was carried out according to a protocol previously described^19^. Briefly, pneumococci were lysed using a solution of 0.1% sodium deoxycholate. Pneumococcal lysates were then added to a 96-well plate containing 4% of sheep blood solution. The plate was incubated for 30 min at 37 °C or until a blood pellet was observed in the wells without lysates. The supernatants were then removed and placed in a fresh 96-well plate in order to determine the levels of haemoglobin released. A toxoid recombinant Ply (PdB)^78^ was used as a negative control. The absorbance was read at an optical density of 540 nm.

### Quantification of cytosolic pneumolysin by ELISA

The determination of pneumolysin concentrations in pneumococcal lysate supernatant, were assessed as previously described, with minor modifications^79^. A 96-well plate was coated with 1 μg of anti-Ply mouse monoclonal antibody (IgG1-Ply4, Abcam Ab71810) per well and incubated overnight, at room temperature. The plate was washed with 200 µl/well of PBS containing 0.05% Tween between incubation steps. The wells were blocked with 20% FBS in PBS, and incubated at 37 °C for 4 h. A volume of 100 µl per well of sample (in triplicate) was added. A standard curve containing various concentrations of purified recombinant Ply (allele 1, D39) was included which was incubated for 1 h at 37 °C. 1 µg of anti-Ply detection antibody (rabbit polyclonal, Abcam Ab71811) in 100 µl of blocking buffer was added to each well, and further incubated for 1 h at 37 °C. A 1:5,000 dilution of goat anti-rabbit IgG alkaline phosphatase (Abcam Ab97048) was then added (100 μl per well), and the plate was incubated for 30 min at 37 °C. Color development was achieved by adding 100 µl/well of para-Nitrophenylphosphate (pNPP) and incubated away from light for 10 min at room temperature. A solution of 1 M of NaOH solution was used to stop the reaction and the concentration of Ply was determined by reading optical density at a wavelength of 405 nm. The D39Δ*ply*-isogenic strain coined PLN-A^80^ was used as negative control.

### Contact-dependent haemolytic assay

Determination of contact-dependent haemolytic activity of ST306, ST615 as well as D39, D39Δ*Ply* and TIGR4 was carried out according to a protocol previously described^59^. In brief, bacterial cell suspensions in PBS (2.0 × 10^9^ CFU/ml) were mixed with equal volumes of 25% sheep red blood cells, centrifuged at 9,600 g for 5 min at 4 °C and incubated for 3 h at 37 °C. No-contact control tubes (no centrifugation) were included. To account for centrifugation-induced lysis of *S. pneumoniae*, bacterial suspensions alone were centrifuged as above, then mixed with erythrocytes and incubated for 3 h at 37 °C. PBS controls (no red blood cells) were also included, and all assays were run in triplicate. Finally, to determine the haemolytic activity of the respective pneumococcal strains tested, the suspensions were briefly centrifuged (9,600 *g*, 1 min, 4 °C) and the supernatant was collected for measurement of absorbance at 490 nm. The D39Δ*ply*-isogenic strain coined PLN-A^80^ was used as negative control and TIGR4 as a positive control.

### Cell wall fractionation and Western blot analysis

For each respective *S. pneumoniae* isolate, a 10 ml liquid culture in Brain Heart Infusion (BHI) medium was incubated overnight until bacteria reached a minimum viable count of 10^8^ CFU/ml. The bacteria were then incubated with 10 μl of penicillin/streptomycin (Sigma Aldrich P4333) for 1 h. The bacterial culture was then pulse-sonicated for 10 s on ice. To dissociate the pneumococcal cell wall from the cytosolic fraction, the culture was span down at 900 g for 20 min, where the supernatant i.e., cytosol content was collected and stored for further analysis. The cell wall pellet was then resuspended in 750 μl of 2% SDS and incubated at 95 °C for 15 min. The cell wall fraction was then centrifuged at 10,000* g* for 20 min at room temperature. The supernatant was removed and analysed separately; the pellet was resuspended in 1 ml of 8 M LiCl and incubated at 37 °C for 15 min. A final centrifugation step was carried out for 30 min at 10,000* g* and the pellet was resuspended in 1 ml of 10 mM EDTA, and further incubated for 15 min. The pellet was centrifuged at 10,000* g* for 30 min, washed with acetone and resuspended in dH2O. To release any cell wall-bound proteins, 10 μl of sodium deoxycholate were added to the mixture to remove all peptidoglycans. 40 μl of both cell wall and cytosolic fractions were loaded onto a 10% SDS-PAGE gel (110 V, 80 min) followed by a wet blot transfer onto nitrocellulose membrane. To test for the presence of pneumolysin, the membrane was incubated with a mouse monoclonal anti-Ply antibody (IgG1-Ply4, Abcam Ab71810) used at a concentration of 3 μg/ml followed by an anti-mouse HRP-conjugated antibody (rabbit polyclonal IgG, New England BioLabs 7076S) at a dilution of 1:2,000.

### Genomics analysis

Twenty-three previously published sequenced whole genomes for *S. pneumoniae* serotype 1 ST306 (n = 17) and ST615 (n = 6) isolates were analysed to determine genetic similarities between the two clones^17^. The ST615 clone is localised in South America and the isolates were from Argentina (n = 5) and Peru (n = 1) while the ST306 clone most common in Europe and the isolates used were from Sweden (n = 14), Spain (n = 1), Qatar (n = 1) and Croatia (n = 1). The sequence reads for the isolates were mapped against a serotype 1 reference genome to generate consensus pseudo-genome sequences and whole genome alignment of the isolates. Mapping was done using SMALT v0.7.4 (Genome Research, Ltd) and realignment of genomic sites with insertions and deletions was done using GATK v3.3^81^. Phylogenetic tree of the strains was constructed based on the recombination-free alignments using RAxML^81^. The reads were also assembled de novo using an automated assembly pipeline^82^. Annotation of the final draft assembled genomes were done using Prokka v1.11^83^. Association of accessory genes with sequence types (ST) or clones was done using Scoary v1.6.16^48^ and genes with Bonferroni corrected *P*-values less than 0.05 were reported. We extracted and translated nucleotide sequences of *Ply* using BLAST^84^ from previously published serotype 1, TIGR4 and D39 genomes to compare the distribution of *Ply* alleles across the serotype 1 population. The Ply amino acid sequences were then aligned and used to construct a phylogenetic tree using FastTree v2.1.7 SSE3^85^. The data is publicly available and each isolate’s ENA accession number is shown in the phylogenetic trees.

### Opsonophagocytosis killing assay (OPKA)

A reference opsonophagocytic assay described elsewhere^86^ was used with minor modifications. Briefly, 5 × 10^4^ DMF-differentiated HL-60 cells were incubated with 5 × 10^2^ opsonised *S. pneumoniae* and rabbit complement, for 45 min at 37 °C/5% CO_2_. IVIG was used at a final dilution of 1:16 as the source for pathogen-specific antibody for opsonisation. Wells containing non-opsonised pneumococci and heat-inactivated complement were used as controls. Viable pneumococcal counts were performed using BAB plates incubated under anaerobic conditions overnight at 37 °C.

### Complement deposition assay

C3 complement deposition on the surface of *S. pneumoniae* was assayed using a previously described procedure^87^ with minor modifications. Bacterial aliquots (10^7^ CFU/ml) were pelleted and resuspended in 100 μl of 20% human serum in PBS, incubated for 30 min at 37 °C, washed and resuspended in 100 μl of monoclonal mouse anti-human C3 antibody (1:300, Abcam). Tubes were further incubated at 37 °C/5% CO2 for 30 min, washed and centrifuged at 17,000 × *g* for 3 min. Pellets were reconstituted in 100 μl of APC-conjugated polyclonal anti-mouse IgG (1:500, Abcam). After incubation on ice for 30 min, the bacteria were washed and resuspended in 500 μl of PBS. Thiazol orange (0.5 μM) was used to detect viable bacterial cells. The tubes were briefly vortexed and incubated for 5 min at room temperature, until acquisition on a flow cytometer (BD FACSCANTO II, BD Biosciences). Mean fluorescence intensity (MFI) was calculated to quantify C3 complement deposition.

### Statistical analysis

For comparison of multiple groups, the statistical significance of endpoints was evaluated by one-way ANOVA followed by Tukey’s multiple comparisons post hoc test. Data normality was tested using the Kolmogorov–Smirnov test. For comparison of two groups, for both the in vivo murine data and SNP diversity plot, the unpaired two-tailed Student’s t-test was used. Data are presented as means ± standard error of the mean (SEM) in bar graphs. Statistical significance was reported by the p-value of the statistical test procedures and was assessed as significant (*, *p* < 0.05), very significant (**, *p* < 0.01), extremely significant (**, *p* < 0.001). All statistical analyses were performed with Prism software version 8.0, GraphPad, Inc.

## Supplementary information

Supplementary Information.
