# Author Correction: Hypervirulent pneumococcal serotype 1 harbours two pneumolysin variants with differential haemolytic activity

**DOI:** 10.1038/s41598-021-86214-1

**Published:** 2021-03-17

**Authors:** Stavros Panagiotou, Chrispin Chaguza, Reham Yahya, Teerawit Audshasai, Murielle Baltazar, Lorenzo Ressel, Shadia Khandaker, Mansoor Alsahag, Tim J. Mitchell, Marc Prudhomme, Aras Kadioglu, Marie Yang

**Affiliations:** 1 Department of Clinical Infection Microbiology and Immunology, Institute of Infection and Global Health, University of Liverpool, The Ronald Ross Building, 8 West Derby St, Liverpool, L69 7BE UK; 2 Wellcome Sanger Institute, Wellcome Genome Campus, Hinxton, Cambridgeshire, CB10 1SA UK; 3 Darwin College, University of Cambridge, Silver Street, Cambridge, CB3 9EU UK; 4 Department of Veterinary Pathology and Public Health, Institute of Veterinary Science, University of Liverpool, Leahurst Campus, Neston, CH64 7TE UK; 5 Institute of Microbiology and Infection, College of Medical and Dental Sciences, University of Birmingham, Birmingham, B15 2TT UK; 6 Université Paul Sabatier, Centre National de La Recherche Scientifique, 118 Route de Narbonne, 31062 Toulouse Cedex 9, France; 7 Faculty of Applied Medical Sciences, Albaha University, Albaha, Kingdom of Saudi Arabia

Correction to: *Scientific Reports* 10.1038/s41598-020-73454-w, published online 14 October 2020

The original version of this Article omitted an affiliation for Mansoor Alsahag. The correct affiliations for Mansoor Alsahag are listed below:

Department of Clinical Infection Microbiology and Immunology, Institute of Infection and Global Health, University of Liverpool, The Ronald Ross Building, 8 West Derby St, Liverpool L69 7BE, UK.

Faculty of Applied Medical Sciences, Albaha University, Albaha, Kingdom of Saudi Arabia.

This has now been corrected in the HTML and PDF versions of this Article, and in the accompanying Supplementary Information file.

