# Author Correction: Hypervirulent pneumococcal serotype 1 harbours two pneumolysin variants with differential haemolytic activity

**DOI:** 10.1038/s41598-022-08813-w

**Published:** 2022-03-24

**Authors:** Stavros Panagiotou, Chrispin Chaguza, Reham Yahya, Teerawit Audshasai, Murielle Baltazar, Lorenzo Ressel, Shadia Khandaker, Mansoor Alsahag, Tim J. Mitchell, Marc Prudhomme, Aras Kadioglu, Marie Yang

**Affiliations:** 1 Department of Clinical Infection Microbiology and Immunology, Institute of Infection and Global Health, University of Liverpool, The Ronald Ross Building, 8 West Derby St, Liverpool, L69 7BE UK; 2 Wellcome Sanger Institute, Wellcome Genome Campus, Hinxton, CB10 1SA Cambridgeshire UK; 3 Darwin College, University of Cambridge, Silver Street, Cambridge, CB3 9EU UK; 4 Department of Veterinary Pathology and Public Health, Institute of Veterinary Science, University of Liverpool, Leahurst Campus, Neston, CH64 7TE UK; 5 Institute of Microbiology and Infection, College of Medical and Dental Sciences, University of Birmingham, Birmingham, B15 2TT UK; 6 Université Paul Sabatier, Centre National de La Recherche Scientifique, 118 Route de Narbonne, 31062 Toulouse Cedex 9, France; 7 Faculty of Applied Medical Sciences, Albaha University, Albaha, Kingdom of Saudi Arabia; 8 College of Sciences and Health Professions, King Saud Bin Abdulaziz University for Health Sciences, Riyadh, Saudi Arabia; 9 King Abdullah International Medical Research Center, Riyadh, Saudi Arabia

Correction to: *Scientific Reports* 10.1038/s41598-020-73454-w, published online 14 October 2020

In the original version of this Article, Reham Yahya was incorrectly affiliated with ‘Department of Clinical Infection Microbiology and Immunology, Institute of Infection and Global Health, University of Liverpool, The Ronald Ross Building, 8 West Derby St, Liverpool L69 7BE, UK.’ The correct affiliations are listed below.

College of sciences and health professions, King Saud bin Abdulaziz University for Health Sciences, Riyadh, Saudi Arabia

King Abdullah International Medical Research Center, Riyadh, Saudi Arabia

The original Article has been corrected.

