# Supplementary material for: Hypervirulent pneumococcal serotype 1 harbours two pneumolysin variants with differential haemolytic activity
Source: Sci Rep. 2020 Oct 14;10:17313. doi: 10.1038/s41598-020-73454-w (PMC7560715; doi:10.1038/s41598-020-73454-w)

Panagiotou et al. 2020 Supplemental Tables

**Hypervirulent pneumococcal serotype 1 harbours two pneumolysin variants with differential haemolytic activity**

Stavros Panagiotou^1^, Chrispin Chaguza^2,3^, Reham Yahya^8,9^, Teerawit Audshasai^1^, Murielle Baltazar^1^, Lorenzo Ressel^4^, Shadia Khandaker^1^, Mansoor Alsahag^1,7^, Tim J. Mitchell^5^, Marc Prudhomme^6^, Aras Kadioglu^1*¶^, Marie Yang^1*¶.^

^1^ Department of Clinical Infection Microbiology & Immunology, Institute of Infection & Global Health, University of Liverpool, The Ronald Ross Building, 8 West Derby St, Liverpool, L69 7BE, UK, ^2^ Wellcome Sanger Institute, Wellcome Genome Campus, Hinxton, Cambridgeshire, CB10 1SA, UK ^3^ University of Cambridge, Darwin College, Silver Street, Cambridge, CB3 9EU, UK, ^4^ Department of Veterinary Pathology and Public Health, Institute of Veterinary Science, University of Liverpool, Leahurst Campus, Neston, CH64 7TE, UK, ^5^Institute of Microbiology and Infection, College of Medical and Dental Sciences University of Birmingham, Birmingham, B15 2TT UK, ^6^ Université Paul Sabatier, Centre National de la Recherche Scientifique, 118 Route de Narbonne 31062 Toulouse Cedex 9, France, ^7^ Faculty of Applied Medical Sciences, Albaha University, Albaha, Kingdom of Saudi Arabia,

^8^ College of sciences and health professions, King Saud bin Abdulaziz University for Health Sciences, Riyadh, Saudi Arabia. ^9^ King Abdullah International Medical Research Center, Riyadh, Saudi Arabia

* Co-senior authors. ^¶^ Corresponding authors: Prof Aras Kadioglu (a.kadioglu@liverpool.ac.uk) and Dr Marie Yang (marie.yang@liverpool.ac.uk)

**Supplemental Tables 1.** Primers designed and used in this study.

| Name | Sequence 5’>3’ |
| --- | --- |
| Ply- Rec-F | GATGAGCGCGACCCAGTGCCAG |
| Ply-Rec-R | GATCCCCAGCTTGCAGACTTGTTCAGCGAAATCAG |
| Ply-Seq-F | TGGATCCTGCTTGAGTTTATCTCTTGCCTAGCG |
| Ply-Seq-R | GGGCTTGTTTAGCACGGTCGATAAC |
| AphA3-Rec-F | CAAGCTGGGGATCCGTTTGA |
| AphA3-Rec-R | TCAAGCAGGATCCATCGATAC |

**Supplemental Tables 2**. **Pneumolysin allele alignment.** The first row refers to amino acid positions. The second row shows allele 1 Ply expressed in S. pneumoniae D39. The amino acid polymorphisms are indicated in bold. Deletion of an amino acid is represented by the abbreviation DEL.

| Serotype | MLST | Allele | Amino acid position | | | | | | |
| --- | --- | --- | --- | --- | --- | --- | --- | --- | --- |
|  |  |  | 150 | 172 | 224 | 265 | 270 | 271 | 380 |
| 2 (D39) | 595 | 1 | Y | T | K | A | V | K | D |
| 1 | 306 | 5 | **H** | **I** | **R** | **S** | **DEL** | **DEL** | D |
| 1 | 615 | 2 | Y | T | K | A | V | K | **N** |
| 4 (TIGR4) | 205 | 2 | Y | T | K | A | V | K | **N** |

**Supplemental Tables 3. List of ST-specific genes and encoded products.** When the genomes of pneumococcal sequence type (ST) 615 vs. ST306 were compared, we found presence and absence differences between isolates in 588 accessory genes (total). After running Scoary, 396 of the genes were found to be significantly associated with a single ST. Amongst them, n=198 i.e., half, were unique to each ST. The list of genes uniquely associated with each respective ST is shown.

| **Gene** | **Gene Length (bp)** | **Gene Presence** | | **Gene Absence** | | **Naïve P-value** | **Bonferroni P-value** | **Benjamini P-value** | **Annotation** |
| --- | --- | --- | --- | --- | --- | --- | --- | --- | --- |
|  |  | **ST306** | **ST615** | **ST306** | **ST615** |  |  |  |  |
| group_816 | 342 | 0 (0/17) | 100 (6/6) | 100 (17/17) | 0 (0/6) | 9.91E-06 | 0.008519322 | 2.15E-05 | integrase core subunit |
| group_773 | 159 | 0 (0/17) | 100 (6/6) | 100 (17/17) | 0 (0/6) | 9.91E-06 | 0.008519322 | 2.15E-05 | transposase |
| group_771 | 597 | 0 (0/17) | 100 (6/6) | 100 (17/17) | 0 (0/6) | 9.91E-06 | 0.008519322 | 2.15E-05 | replication initiator protein A N-terminus |
| group_770 | 381 | 0 (0/17) | 100 (6/6) | 100 (17/17) | 0 (0/6) | 9.91E-06 | 0.008519322 | 2.15E-05 | arsenate reductase-like glutaredoxin family protein |
| group_812 | 183 | 0 (0/17) | 100 (6/6) | 100 (17/17) | 0 (0/6) | 9.91E-06 | 0.008519322 | 2.15E-05 | methyltransferase small domain superfamily protein |
| group_813 | 327 | 0 (0/17) | 100 (6/6) | 100 (17/17) | 0 (0/6) | 9.91E-06 | 0.008519322 | 2.15E-05 | competence protein ComGC |
| group_775 | 195 | 0 (0/17) | 100 (6/6) | 100 (17/17) | 0 (0/6) | 9.91E-06 | 0.008519322 | 2.15E-05 | hypothetical protein |
| group_774 | 192 | 0 (0/17) | 100 (6/6) | 100 (17/17) | 0 (0/6) | 9.91E-06 | 0.008519322 | 2.15E-05 | alanine aminotransferase |
| group_952 | 324 | 100 (17/17) | 0 (0/6) | 0 (0/17) | 100 (6/6) | 9.91E-06 | 0.008519322 | 2.15E-05 | hypothetical protein |
| group_818 | 690 | 0 (0/17) | 100 (6/6) | 100 (17/17) | 0 (0/6) | 9.91E-06 | 0.008519322 | 2.15E-05 | phage repressor-like protein |
| group_171 | 339 | 0 (0/17) | 100 (6/6) | 100 (17/17) | 0 (0/6) | 9.91E-06 | 0.008519322 | 2.15E-05 | 3-ketoacyl-ACP reductase |
| group_172 | 348 | 0 (0/17) | 100 (6/6) | 100 (17/17) | 0 (0/6) | 9.91E-06 | 0.008519322 | 2.15E-05 | chlorohydrolase |
| group_414 | 141 | 0 (0/17) | 100 (6/6) | 100 (17/17) | 0 (0/6) | 9.91E-06 | 0.008519322 | 2.15E-05 | hypothetical protein |
| group_177 | 456 | 0 (0/17) | 100 (6/6) | 100 (17/17) | 0 (0/6) | 9.91E-06 | 0.008519322 | 2.15E-05 | transposase |
| group_176 | 276 | 100 (17/17) | 0 (0/6) | 0 (0/17) | 100 (6/6) | 9.91E-06 | 0.008519322 | 2.15E-05 | degenerate transposase |
| group_1063 | 300 | 100 (17/17) | 0 (0/6) | 0 (0/17) | 100 (6/6) | 9.91E-06 | 0.008519322 | 2.15E-05 | hypothetical protein |
| group_777 | 738 | 0 (0/17) | 100 (6/6) | 100 (17/17) | 0 (0/6) | 9.91E-06 | 0.008519322 | 2.15E-05 | multidrug ABC transporter permease |
| group_1061 | 726 | 100 (17/17) | 0 (0/6) | 0 (0/17) | 100 (6/6) | 9.91E-06 | 0.008519322 | 2.15E-05 | hypothetical protein |
| group_1060 | 738 | 100 (17/17) | 0 (0/6) | 0 (0/17) | 100 (6/6) | 9.91E-06 | 0.008519322 | 2.15E-05 | Uncharacterized protein conserved in bacteria |
| group_1066 | 360 | 100 (17/17) | 0 (0/6) | 0 (0/17) | 100 (6/6) | 9.91E-06 | 0.008519322 | 2.15E-05 | hypothetical protein |
| group_1065 | 165 | 100 (17/17) | 0 (0/6) | 0 (0/17) | 100 (6/6) | 9.91E-06 | 0.008519322 | 2.15E-05 | hypothetical protein |
| group_1064 | 771 | 100 (17/17) | 0 (0/6) | 0 (0/17) | 100 (6/6) | 9.91E-06 | 0.008519322 | 2.15E-05 | Streptolysin S biosynthesis protein |
| ybhF | 885 | 0 (0/17) | 100 (6/6) | 100 (17/17) | 0 (0/6) | 9.91E-06 | 0.008519322 | 2.15E-05 | ABC transporter ATP-binding protein |
| group_889 | 588 | 100 (17/17) | 0 (0/6) | 0 (0/17) | 100 (6/6) | 9.91E-06 | 0.008519322 | 2.15E-05 | Tn5253 CAAX amino terminal protease family |
| group_888 | 2610 | 100 (17/17) | 0 (0/6) | 0 (0/17) | 100 (6/6) | 9.91E-06 | 0.008519322 | 2.15E-05 | lantibiotic synthetase |
| group_884 | 1320 | 100 (17/17) | 0 (0/6) | 0 (0/17) | 100 (6/6) | 9.91E-06 | 0.008519322 | 2.15E-05 | permease |
| group_881 | 609 | 100 (17/17) | 0 (0/6) | 0 (0/17) | 100 (6/6) | 9.91E-06 | 0.008519322 | 2.15E-05 | Predicted integral membrane protein |
| group_883 | 537 | 100 (17/17) | 0 (0/6) | 0 (0/17) | 100 (6/6) | 9.91E-06 | 0.008519322 | 2.15E-05 | IS861 transposase Orf1 |
| group_882 | 840 | 100 (17/17) | 0 (0/6) | 0 (0/17) | 100 (6/6) | 9.91E-06 | 0.008519322 | 2.15E-05 | IS861 transposase Orf2 |
| pgpA | 489 | 0 (0/17) | 100 (6/6) | 100 (17/17) | 0 (0/6) | 9.91E-06 | 0.008519322 | 2.15E-05 | phosphatidylglycerophosphatase A |
| fbpC_2 | 267 | 100 (17/17) | 0 (0/6) | 0 (0/17) | 100 (6/6) | 9.91E-06 | 0.008519322 | 2.15E-05 | ABC transporter ATP-binding protein |
| transposase_D_2 | 414 | 100 (17/17) | 0 (0/6) | 0 (0/17) | 100 (6/6) | 9.91E-06 | 0.008519322 | 2.15E-05 | degenerate transposase |
| manZ_4 | 801 | 100 (17/17) | 0 (0/6) | 0 (0/17) | 100 (6/6) | 9.91E-06 | 0.008519322 | 2.15E-05 | PTS system mannose/fructose/N-acetylgalactosamine-specific transporter subunit IID |
| mga2 | 1485 | 100 (17/17) | 0 (0/6) | 0 (0/17) | 100 (6/6) | 9.91E-06 | 0.008519322 | 2.15E-05 | M protein trans-acting positive transcriptional regulator |
| group_926 | 138 | 100 (17/17) | 0 (0/6) | 0 (0/17) | 100 (6/6) | 9.91E-06 | 0.008519322 | 2.15E-05 | hypothetical protein |
| nanM | 924 | 0 (0/17) | 100 (6/6) | 100 (17/17) | 0 (0/6) | 9.91E-06 | 0.008519322 | 2.15E-05 | N-acetylneuraminate epimerase precursor |
| yeiH | 1011 | 100 (17/17) | 0 (0/6) | 0 (0/17) | 100 (6/6) | 9.91E-06 | 0.008519322 | 2.15E-05 | membrane protein |
| group_927 | 915 | 100 (17/17) | 0 (0/6) | 0 (0/17) | 100 (6/6) | 9.91E-06 | 0.008519322 | 2.15E-05 | HesA/MoeB/ThiF family protein |
| yqbO | 2760 | 0 (0/17) | 100 (6/6) | 100 (17/17) | 0 (0/6) | 9.91E-06 | 0.008519322 | 2.15E-05 | prophage LambdaSa04, tail tape measure protein, TP901 family |
| group_228 | 507 | 100 (17/17) | 0 (0/6) | 0 (0/17) | 100 (6/6) | 9.91E-06 | 0.008519322 | 2.15E-05 | ABC transporter ATPase |
| group_497 | 244 | 0 (0/17) | 100 (6/6) | 100 (17/17) | 0 (0/6) | 9.91E-06 | 0.008519322 | 2.15E-05 | hypothetical protein |
| group_1062 | 807 | 100 (17/17) | 0 (0/6) | 0 (0/17) | 100 (6/6) | 9.91E-06 | 0.008519322 | 2.15E-05 | sodium-dependent transporter |
| group_650 | 354 | 0 (0/17) | 100 (6/6) | 100 (17/17) | 0 (0/6) | 9.91E-06 | 0.008519322 | 2.15E-05 | hypothetical protein |
| group_651 | 684 | 0 (0/17) | 100 (6/6) | 100 (17/17) | 0 (0/6) | 9.91E-06 | 0.008519322 | 2.15E-05 | molecular chaperone |
| group_655 | 339 | 0 (0/17) | 100 (6/6) | 100 (17/17) | 0 (0/6) | 9.91E-06 | 0.008519322 | 2.15E-05 | antibiotic biosynthesis monooxygenase subfamily protein |
| group_657 | 435 | 0 (0/17) | 100 (6/6) | 100 (17/17) | 0 (0/6) | 9.91E-06 | 0.008519322 | 2.15E-05 | caax amino protease family protein |
| group_790 | 1158 | 0 (0/17) | 100 (6/6) | 100 (17/17) | 0 (0/6) | 9.91E-06 | 0.008519322 | 2.15E-05 | phosphoesterase |
| group_793 | 705 | 0 (0/17) | 100 (6/6) | 100 (17/17) | 0 (0/6) | 9.91E-06 | 0.008519322 | 2.15E-05 | truncated MobA family endonuclease relaxase |
| group_792 | 228 | 0 (0/17) | 100 (6/6) | 100 (17/17) | 0 (0/6) | 9.91E-06 | 0.008519322 | 2.15E-05 | MobC family protein |
| group_795 | 513 | 0 (0/17) | 100 (6/6) | 100 (17/17) | 0 (0/6) | 9.91E-06 | 0.008519322 | 2.15E-05 | MutR family transcriptional regulator |
| group_794 | 384 | 0 (0/17) | 100 (6/6) | 100 (17/17) | 0 (0/6) | 9.91E-06 | 0.008519322 | 2.15E-05 | transcriptional activator, Rgg/GadR/MutR family protein |
| zmpA | 6420 | 0 (0/17) | 100 (6/6) | 100 (17/17) | 0 (0/6) | 9.91E-06 | 0.008519322 | 2.15E-05 | IgA-protease |
| group_796 | 831 | 0 (0/17) | 100 (6/6) | 100 (17/17) | 0 (0/6) | 9.91E-06 | 0.008519322 | 2.15E-05 | NAD binding domain of 6-phosphogluconate dehydrogenase family |
| malX_1 | 1293 | 0 (0/17) | 100 (6/6) | 100 (17/17) | 0 (0/6) | 9.91E-06 | 0.008519322 | 2.15E-05 | bacterial extracellular solute-binding protein |
| ABC-NBD-truncation_2 | 654 | 100 (17/17) | 0 (0/6) | 0 (0/17) | 100 (6/6) | 9.91E-06 | 0.008519322 | 2.15E-05 | ABC transporter ATP-binding protein |
| group_1930 | 123 | 0 (0/17) | 100 (6/6) | 100 (17/17) | 0 (0/6) | 9.91E-06 | 0.008519322 | 2.15E-05 | degenerate transposase |
| group_679 | 744 | 0 (0/17) | 100 (6/6) | 100 (17/17) | 0 (0/6) | 9.91E-06 | 0.008519322 | 2.15E-05 | 16S ribosomal RNA methyltransferase RsmE |
| group_928 | 1014 | 100 (17/17) | 0 (0/6) | 0 (0/17) | 100 (6/6) | 9.91E-06 | 0.008519322 | 2.15E-05 | hypothetical protein |
| oppC | 852 | 0 (0/17) | 100 (6/6) | 100 (17/17) | 0 (0/6) | 9.91E-06 | 0.008519322 | 2.15E-05 | oligopeptide ABC transporter pemease protein |
| rafA | 2217 | 0 (0/17) | 100 (6/6) | 100 (17/17) | 0 (0/6) | 9.91E-06 | 0.008519322 | 2.15E-05 | alpha-galactosidase AgaN |
| group_1023 | 198 | 100 (17/17) | 0 (0/6) | 0 (0/17) | 100 (6/6) | 9.91E-06 | 0.008519322 | 2.15E-05 | transcriptional regulator |
| group_1022 | 867 | 100 (17/17) | 0 (0/6) | 0 (0/17) | 100 (6/6) | 9.91E-06 | 0.008519322 | 2.15E-05 | transcriptional regulator |
| group_1021 | 129 | 100 (17/17) | 0 (0/6) | 0 (0/17) | 100 (6/6) | 9.91E-06 | 0.008519322 | 2.15E-05 | hypothetical protein |
| group_960 | 774 | 100 (17/17) | 0 (0/6) | 0 (0/17) | 100 (6/6) | 9.91E-06 | 0.008519322 | 2.15E-05 | Ser/Thr protein phosphatase family protein |
| group_728 | 144 | 0 (0/17) | 100 (6/6) | 100 (17/17) | 0 (0/6) | 9.91E-06 | 0.008519322 | 2.15E-05 | hypothetical protein |
| group_729 | 237 | 0 (0/17) | 100 (6/6) | 100 (17/17) | 0 (0/6) | 9.91E-06 | 0.008519322 | 2.15E-05 | gp29 |
| group_720 | 576 | 0 (0/17) | 100 (6/6) | 100 (17/17) | 0 (0/6) | 9.91E-06 | 0.008519322 | 2.15E-05 | bacteriophage maturation protease |
| group_721 | 1203 | 0 (0/17) | 100 (6/6) | 100 (17/17) | 0 (0/6) | 9.91E-06 | 0.008519322 | 2.15E-05 | portal protein |
| group_722 | 219 | 0 (0/17) | 100 (6/6) | 100 (17/17) | 0 (0/6) | 9.91E-06 | 0.008519322 | 2.15E-05 | hypothetical protein |
| group_723 | 1731 | 0 (0/17) | 100 (6/6) | 100 (17/17) | 0 (0/6) | 9.91E-06 | 0.008519322 | 2.15E-05 | bacteriophage terminase |
| group_724 | 393 | 0 (0/17) | 100 (6/6) | 100 (17/17) | 0 (0/6) | 9.91E-06 | 0.008519322 | 2.15E-05 | bacteriophage terminase protein, small subunit |
| group_725 | 306 | 0 (0/17) | 100 (6/6) | 100 (17/17) | 0 (0/6) | 9.91E-06 | 0.008519322 | 2.15E-05 | bacteriophage nuclease |
| group_726 | 591 | 0 (0/17) | 100 (6/6) | 100 (17/17) | 0 (0/6) | 9.91E-06 | 0.008519322 | 2.15E-05 | prophage LambdaSa2, site-specific recombinase, phage integrase family |
| group_727 | 402 | 0 (0/17) | 100 (6/6) | 100 (17/17) | 0 (0/6) | 9.91E-06 | 0.008519322 | 2.15E-05 | bacteriophage transcriptional activator |
| iga | 5643 | 0 (0/17) | 100 (6/6) | 100 (17/17) | 0 (0/6) | 9.91E-06 | 0.008519322 | 2.15E-05 | zinc metalloprotease ZmpB |
| group_1056 | 909 | 100 (17/17) | 0 (0/6) | 0 (0/17) | 100 (6/6) | 9.91E-06 | 0.008519322 | 2.15E-05 | hypothetical protein |
| group_1057 | 762 | 100 (17/17) | 0 (0/6) | 0 (0/17) | 100 (6/6) | 9.91E-06 | 0.008519322 | 2.15E-05 | hypothetical protein |
| group_1055 | 1899 | 100 (17/17) | 0 (0/6) | 0 (0/17) | 100 (6/6) | 9.91E-06 | 0.008519322 | 2.15E-05 | YcaO-like family protein |
| group_1058 | 171 | 100 (17/17) | 0 (0/6) | 0 (0/17) | 100 (6/6) | 9.91E-06 | 0.008519322 | 2.15E-05 | ABC transporter ATP-binding protein |
| group_782 | 327 | 0 (0/17) | 100 (6/6) | 100 (17/17) | 0 (0/6) | 9.91E-06 | 0.008519322 | 2.15E-05 | hypothetical cytosolic protein |
| group_919 | 714 | 100 (17/17) | 0 (0/6) | 0 (0/17) | 100 (6/6) | 9.91E-06 | 0.008519322 | 2.15E-05 | ABC transporter permease subunit |
| group_913 | 123 | 100 (17/17) | 0 (0/6) | 0 (0/17) | 100 (6/6) | 9.91E-06 | 0.008519322 | 2.15E-05 | hypothetical protein |
| group_917 | 162 | 100 (17/17) | 0 (0/6) | 0 (0/17) | 100 (6/6) | 9.91E-06 | 0.008519322 | 2.15E-05 | hypothetical protein |
| group_780 | 189 | 0 (0/17) | 100 (6/6) | 100 (17/17) | 0 (0/6) | 9.91E-06 | 0.008519322 | 2.15E-05 | hypothetical protein |
| group_915 | 375 | 100 (17/17) | 0 (0/6) | 0 (0/17) | 100 (6/6) | 9.91E-06 | 0.008519322 | 2.15E-05 | Peptidase |
| group_914 | 456 | 100 (17/17) | 0 (0/6) | 0 (0/17) | 100 (6/6) | 9.91E-06 | 0.008519322 | 2.15E-05 | Peptidase |
| group_781 | 231 | 0 (0/17) | 100 (6/6) | 100 (17/17) | 0 (0/6) | 9.91E-06 | 0.008519322 | 2.15E-05 | MarR family transcriptional regulator |
| group_618 | 1380 | 100 (17/17) | 0 (0/6) | 0 (0/17) | 100 (6/6) | 9.91E-06 | 0.008519322 | 2.15E-05 | McrBC 5-methylcytosine restriction system component |
| group_619 | 729 | 100 (17/17) | 0 (0/6) | 0 (0/17) | 100 (6/6) | 9.91E-06 | 0.008519322 | 2.15E-05 | GNAT family acetyltransferase |
| group_616 | 252 | 100 (17/17) | 0 (0/6) | 0 (0/17) | 100 (6/6) | 9.91E-06 | 0.008519322 | 2.15E-05 | IS66-Spn1 transposase |
| group_347 | 615 | 100 (17/17) | 0 (0/6) | 0 (0/17) | 100 (6/6) | 9.91E-06 | 0.008519322 | 2.15E-05 | Type I restriction modification system protein |
| spxA_1 | 381 | 100 (17/17) | 0 (0/6) | 0 (0/17) | 100 (6/6) | 9.91E-06 | 0.008519322 | 2.15E-05 | arsenate reductase-like glutaredoxin family protein |
| fucA | 639 | 100 (17/17) | 0 (0/6) | 0 (0/17) | 100 (6/6) | 9.91E-06 | 0.008519322 | 2.15E-05 | L-fuculose phosphate aldolase |
| fucK | 1404 | 100 (17/17) | 0 (0/6) | 0 (0/17) | 100 (6/6) | 9.91E-06 | 0.008519322 | 2.15E-05 | L-fuculose kinase fucK |
| group_298 | 288 | 0 (0/17) | 100 (6/6) | 100 (17/17) | 0 (0/6) | 9.91E-06 | 0.008519322 | 2.15E-05 | transposase, ISSmi4 |
| group_299 | 702 | 100 (17/17) | 0 (0/6) | 0 (0/17) | 100 (6/6) | 9.91E-06 | 0.008519322 | 2.15E-05 | transporter major facilitator family protein |
| fucU | 441 | 100 (17/17) | 0 (0/6) | 0 (0/17) | 100 (6/6) | 9.91E-06 | 0.008519322 | 2.15E-05 | fucose operon FucU protein |
| group_965 | 324 | 100 (17/17) | 0 (0/6) | 0 (0/17) | 100 (6/6) | 9.91E-06 | 0.008519322 | 2.15E-05 | hypothetical protein |
| group_290 | 471 | 0 (0/17) | 100 (6/6) | 100 (17/17) | 0 (0/6) | 9.91E-06 | 0.008519322 | 2.15E-05 | choline binding protein G |
| group_297 | 288 | 0 (0/17) | 100 (6/6) | 100 (17/17) | 0 (0/6) | 9.91E-06 | 0.008519322 | 2.15E-05 | IS66-Spn1 transposase |
| bglK_2 | 891 | 100 (17/17) | 0 (0/6) | 0 (0/17) | 100 (6/6) | 9.91E-06 | 0.008519322 | 2.15E-05 | transcriptional regulator |
| macB_6 | 642 | 0 (0/17) | 100 (6/6) | 100 (17/17) | 0 (0/6) | 9.91E-06 | 0.008519322 | 2.15E-05 | ABC transporter ATP-binding protein |
| manY_2 | 774 | 100 (17/17) | 0 (0/6) | 0 (0/17) | 100 (6/6) | 9.91E-06 | 0.008519322 | 2.15E-05 | PTS system mannose/fructose/N-acetylgalactosamine-specific transporter subunit IIC |
| glnQ_4 | 885 | 100 (17/17) | 0 (0/6) | 0 (0/17) | 100 (6/6) | 9.91E-06 | 0.008519322 | 2.15E-05 | ABC transporter ATP-binding protein |
| group_669 | 315 | 0 (0/17) | 100 (6/6) | 100 (17/17) | 0 (0/6) | 9.91E-06 | 0.008519322 | 2.15E-05 | hypothetical protein |
| group_668 | 1680 | 0 (0/17) | 100 (6/6) | 100 (17/17) | 0 (0/6) | 9.91E-06 | 0.008519322 | 2.15E-05 | FucA |
| group_660 | 1404 | 0 (0/17) | 100 (6/6) | 100 (17/17) | 0 (0/6) | 9.91E-06 | 0.008519322 | 2.15E-05 | L-fuculose kinase fucK |
| group_663 | 840 | 0 (0/17) | 100 (6/6) | 100 (17/17) | 0 (0/6) | 9.91E-06 | 0.008519322 | 2.15E-05 | sugar ABC transporter permease |
| group_662 | 930 | 0 (0/17) | 100 (6/6) | 100 (17/17) | 0 (0/6) | 9.91E-06 | 0.008519322 | 2.15E-05 | sugar ABC transporter permease |
| group_665 | 1872 | 0 (0/17) | 100 (6/6) | 100 (17/17) | 0 (0/6) | 9.91E-06 | 0.008519322 | 2.15E-05 | F5/8 type C domain containing protein |
| group_664 | 1320 | 0 (0/17) | 100 (6/6) | 100 (17/17) | 0 (0/6) | 9.91E-06 | 0.008519322 | 2.15E-05 | alpha-L-fucosidase |
| group_667 | 729 | 0 (0/17) | 100 (6/6) | 100 (17/17) | 0 (0/6) | 9.91E-06 | 0.008519322 | 2.15E-05 | L-fuculose phosphate aldolase |
| cdd2 | 408 | 0 (0/17) | 100 (6/6) | 100 (17/17) | 0 (0/6) | 9.91E-06 | 0.008519322 | 2.15E-05 | cytidine deaminase |
| levE | 471 | 100 (17/17) | 0 (0/6) | 0 (0/17) | 100 (6/6) | 9.91E-06 | 0.008519322 | 2.15E-05 | PTS system transporter subunit IIB |
| lmrA_1 | 1608 | 100 (17/17) | 0 (0/6) | 0 (0/17) | 100 (6/6) | 9.91E-06 | 0.008519322 | 2.15E-05 | drug efflux ABC transporter ATP-binding/permease protein |
| artP_1 | 807 | 0 (0/17) | 100 (6/6) | 100 (17/17) | 0 (0/6) | 9.91E-06 | 0.008519322 | 2.15E-05 | amino acid ABC transporter periplasmic amino acid-binding protein |
| pncG | 165 | 100 (17/17) | 0 (0/6) | 0 (0/17) | 100 (6/6) | 9.91E-06 | 0.008519322 | 2.15E-05 | putative immunity protein |
| nanB_2 | 2223 | 100 (17/17) | 0 (0/6) | 0 (0/17) | 100 (6/6) | 9.91E-06 | 0.008519322 | 2.15E-05 | sialidase B |
| iga_1 | 5892 | 100 (17/17) | 0 (0/6) | 0 (0/17) | 100 (6/6) | 9.91E-06 | 0.008519322 | 2.15E-05 | immunoglobulin A1 protease |
| group_325 | 555 | 0 (0/17) | 100 (6/6) | 100 (17/17) | 0 (0/6) | 9.91E-06 | 0.008519322 | 2.15E-05 | hypothetical protein |
| ntpI_1 | 1992 | 100 (17/17) | 0 (0/6) | 0 (0/17) | 100 (6/6) | 9.91E-06 | 0.008519322 | 2.15E-05 | V-type H+-ATPase, subunit I |
| group_764 | 123 | 0 (0/17) | 100 (6/6) | 100 (17/17) | 0 (0/6) | 9.91E-06 | 0.008519322 | 2.15E-05 | O-acetylhomoserine sulfhydrylase |
| group_941 | 183 | 100 (17/17) | 0 (0/6) | 0 (0/17) | 100 (6/6) | 9.91E-06 | 0.008519322 | 2.15E-05 | degenerate transposase |
| group_766 | 222 | 0 (0/17) | 100 (6/6) | 100 (17/17) | 0 (0/6) | 9.91E-06 | 0.008519322 | 2.15E-05 | Tn5253 bacteriocine putative |
| group_943 | 588 | 100 (17/17) | 0 (0/6) | 0 (0/17) | 100 (6/6) | 9.91E-06 | 0.008519322 | 2.15E-05 | Tn5253 CAAX amino terminal protease family |
| group_760 | 171 | 0 (0/17) | 100 (6/6) | 100 (17/17) | 0 (0/6) | 9.91E-06 | 0.008519322 | 2.15E-05 | translation factor |
| group_761 | 1344 | 0 (0/17) | 100 (6/6) | 100 (17/17) | 0 (0/6) | 9.91E-06 | 0.008519322 | 2.15E-05 | McrBC 5-methylcytosine restriction system component |
| group_762 | 1278 | 0 (0/17) | 100 (6/6) | 100 (17/17) | 0 (0/6) | 9.91E-06 | 0.008519322 | 2.15E-05 | cell division protein FtsY |
| group_763 | 468 | 0 (0/17) | 100 (6/6) | 100 (17/17) | 0 (0/6) | 9.91E-06 | 0.008519322 | 2.15E-05 | MutT/nudix family protein |
| group_948 | 459 | 100 (17/17) | 0 (0/6) | 0 (0/17) | 100 (6/6) | 9.91E-06 | 0.008519322 | 2.15E-05 | beta-galactosidase subunit beta |
| group_768 | 591 | 0 (0/17) | 100 (6/6) | 100 (17/17) | 0 (0/6) | 9.91E-06 | 0.008519322 | 2.15E-05 | Tn5253 hypothetical protein |
| group_769 | 588 | 0 (0/17) | 100 (6/6) | 100 (17/17) | 0 (0/6) | 9.91E-06 | 0.008519322 | 2.15E-05 | Tn5253 CAAX amino terminal protease family |
| group_321 | 375 | 0 (0/17) | 100 (6/6) | 100 (17/17) | 0 (0/6) | 9.91E-06 | 0.008519322 | 2.15E-05 | ABC transporter |
| group_1018 | 1392 | 100 (17/17) | 0 (0/6) | 0 (0/17) | 100 (6/6) | 9.91E-06 | 0.008519322 | 2.15E-05 | hypothetical protein |
| group_1016 | 864 | 100 (17/17) | 0 (0/6) | 0 (0/17) | 100 (6/6) | 9.91E-06 | 0.008519322 | 2.15E-05 | helix-turn-helix domain protein |
| group_1017 | 1179 | 100 (17/17) | 0 (0/6) | 0 (0/17) | 100 (6/6) | 9.91E-06 | 0.008519322 | 2.15E-05 | Permease |
| group_1014 | 360 | 100 (17/17) | 0 (0/6) | 0 (0/17) | 100 (6/6) | 9.91E-06 | 0.008519322 | 2.15E-05 | hypothetical protein |
| group_1013 | 213 | 100 (17/17) | 0 (0/6) | 0 (0/17) | 100 (6/6) | 9.91E-06 | 0.008519322 | 2.15E-05 | hypothetical protein |
| group_1010 | 183 | 100 (17/17) | 0 (0/6) | 0 (0/17) | 100 (6/6) | 9.91E-06 | 0.008519322 | 2.15E-05 | transcriptional regulator |
| group_1011 | 495 | 100 (17/17) | 0 (0/6) | 0 (0/17) | 100 (6/6) | 9.91E-06 | 0.008519322 | 2.15E-05 | Phage transcriptional regulator |
| group_787 | 456 | 0 (0/17) | 100 (6/6) | 100 (17/17) | 0 (0/6) | 9.91E-06 | 0.008519322 | 2.15E-05 | hypothetical protein |
| group_897 | 123 | 100 (17/17) | 0 (0/6) | 0 (0/17) | 100 (6/6) | 9.91E-06 | 0.008519322 | 2.15E-05 | O-acetylhomoserine sulfhydrylase |
| group_894 | 327 | 100 (17/17) | 0 (0/6) | 0 (0/17) | 100 (6/6) | 9.91E-06 | 0.008519322 | 2.15E-05 | transposase |
| group_892 | 675 | 100 (17/17) | 0 (0/6) | 0 (0/17) | 100 (6/6) | 9.91E-06 | 0.008519322 | 2.15E-05 | ABC transporter membrane protein |
| group_893 | 351 | 100 (17/17) | 0 (0/6) | 0 (0/17) | 100 (6/6) | 9.91E-06 | 0.008519322 | 2.15E-05 | transposase, ISSmi4 |
| group_891 | 255 | 100 (17/17) | 0 (0/6) | 0 (0/17) | 100 (6/6) | 9.91E-06 | 0.008519322 | 2.15E-05 | replication initiator protein |
| group_691 | 1218 | 0 (0/17) | 100 (6/6) | 100 (17/17) | 0 (0/6) | 9.91E-06 | 0.008519322 | 2.15E-05 | preprotein translocase subunit SecY |
| ftsY | 1221 | 100 (17/17) | 0 (0/6) | 0 (0/17) | 100 (6/6) | 9.91E-06 | 0.008519322 | 2.15E-05 | cell division protein FtsY |
| ybbH_1 | 612 | 100 (17/17) | 0 (0/6) | 0 (0/17) | 100 (6/6) | 9.91E-06 | 0.008519322 | 2.15E-05 | phosphosugar-binding transcriptional regulator |
| secY_1 | 1218 | 100 (17/17) | 0 (0/6) | 0 (0/17) | 100 (6/6) | 9.91E-06 | 0.008519322 | 2.15E-05 | preprotein translocase subunit SecY |
| rnhB | 780 | 100 (17/17) | 0 (0/6) | 0 (0/17) | 100 (6/6) | 9.91E-06 | 0.008519322 | 2.15E-05 | ribonuclease HII |
| group_710 | 723 | 0 (0/17) | 100 (6/6) | 100 (17/17) | 0 (0/6) | 9.91E-06 | 0.008519322 | 2.15E-05 | prophage LambdaSa04, tail protein |
| group_713 | 579 | 0 (0/17) | 100 (6/6) | 100 (17/17) | 0 (0/6) | 9.91E-06 | 0.008519322 | 2.15E-05 | bacteriophage pi2 protein 39 |
| group_712 | 420 | 0 (0/17) | 100 (6/6) | 100 (17/17) | 0 (0/6) | 9.91E-06 | 0.008519322 | 2.15E-05 | bacteriophage pi2 protein 40 |
| group_715 | 348 | 0 (0/17) | 100 (6/6) | 100 (17/17) | 0 (0/6) | 9.91E-06 | 0.008519322 | 2.15E-05 | bacteriophage pi2 protein 37 |
| group_714 | 324 | 0 (0/17) | 100 (6/6) | 100 (17/17) | 0 (0/6) | 9.91E-06 | 0.008519322 | 2.15E-05 | bacteriophage pi2 protein 38 |
| group_717 | 282 | 0 (0/17) | 100 (6/6) | 100 (17/17) | 0 (0/6) | 9.91E-06 | 0.008519322 | 2.15E-05 | bacteriophage pi2 protein 35 |
| group_716 | 300 | 0 (0/17) | 100 (6/6) | 100 (17/17) | 0 (0/6) | 9.91E-06 | 0.008519322 | 2.15E-05 | bacteriophage pi2 protein 36 |
| group_719 | 1173 | 0 (0/17) | 100 (6/6) | 100 (17/17) | 0 (0/6) | 9.91E-06 | 0.008519322 | 2.15E-05 | bacteriophage capsid protein |
| group_718 | 291 | 0 (0/17) | 100 (6/6) | 100 (17/17) | 0 (0/6) | 9.91E-06 | 0.008519322 | 2.15E-05 | phage protein |
| group_748 | 204 | 0 (0/17) | 100 (6/6) | 100 (17/17) | 0 (0/6) | 9.91E-06 | 0.008519322 | 2.15E-05 | phage protein |
| group_802 | 1245 | 0 (0/17) | 100 (6/6) | 100 (17/17) | 0 (0/6) | 9.91E-06 | 0.008519322 | 2.15E-05 | biotin carboxylase |
| group_747 | 198 | 0 (0/17) | 100 (6/6) | 100 (17/17) | 0 (0/6) | 9.91E-06 | 0.008519322 | 2.15E-05 | gp9 |
| group_821 | 897 | 0 (0/17) | 100 (6/6) | 100 (17/17) | 0 (0/6) | 9.91E-06 | 0.008519322 | 2.15E-05 | hypothetical protein |
| group_820 | 357 | 0 (0/17) | 100 (6/6) | 100 (17/17) | 0 (0/6) | 9.91E-06 | 0.008519322 | 2.15E-05 | hypothetical protein |
| group_739 | 159 | 0 (0/17) | 100 (6/6) | 100 (17/17) | 0 (0/6) | 9.91E-06 | 0.008519322 | 2.15E-05 | hypothetical protein |
| group_745 | 426 | 0 (0/17) | 100 (6/6) | 100 (17/17) | 0 (0/6) | 9.91E-06 | 0.008519322 | 2.15E-05 | bacteriophage protein |
| group_742 | 321 | 0 (0/17) | 100 (6/6) | 100 (17/17) | 0 (0/6) | 9.91E-06 | 0.008519322 | 2.15E-05 | hypothetical protein |
| group_964 | 234 | 100 (17/17) | 0 (0/6) | 0 (0/17) | 100 (6/6) | 9.91E-06 | 0.008519322 | 2.15E-05 | adenylosuccinate lyase |
| group_231 | 780 | 0 (0/17) | 100 (6/6) | 100 (17/17) | 0 (0/6) | 9.91E-06 | 0.008519322 | 2.15E-05 | ABC transporter ATPase |
| group_620 | 465 | 100 (17/17) | 0 (0/6) | 0 (0/17) | 100 (6/6) | 9.91E-06 | 0.008519322 | 2.15E-05 | hypothetical protein |
| tnp | 360 | 0 (0/17) | 100 (6/6) | 100 (17/17) | 0 (0/6) | 9.91E-06 | 0.008519322 | 2.15E-05 | Tn5252 |
| pcpA | 1866 | 100 (17/17) | 0 (0/6) | 0 (0/17) | 100 (6/6) | 9.91E-06 | 0.008519322 | 2.15E-05 | choline binding protein PcpA |
| argG | 420 | 0 (0/17) | 100 (6/6) | 100 (17/17) | 0 (0/6) | 9.91E-06 | 0.008519322 | 2.15E-05 | argininosuccinate synthase, truncation |
| ydiC | 684 | 100 (17/17) | 0 (0/6) | 0 (0/17) | 100 (6/6) | 9.91E-06 | 0.008519322 | 2.15E-05 | molecular chaperone |
| nanE_2 | 702 | 100 (17/17) | 0 (0/6) | 0 (0/17) | 100 (6/6) | 9.91E-06 | 0.008519322 | 2.15E-05 | N-acetylmannosamine-6-phosphate epimerase |
| nanE_1 | 687 | 100 (17/17) | 0 (0/6) | 0 (0/17) | 100 (6/6) | 9.91E-06 | 0.008519322 | 2.15E-05 | N-acetylmannosamine-6-phosphate 2-epimerase |
| bglH_1 | 282 | 100 (17/17) | 0 (0/6) | 0 (0/17) | 100 (6/6) | 9.91E-06 | 0.008519322 | 2.15E-05 | glycosyl hydrolase |
| ydaF_5 | 471 | 100 (17/17) | 0 (0/6) | 0 (0/17) | 100 (6/6) | 9.91E-06 | 0.008519322 | 2.15E-05 | putative acetyltransferase |
| group_993 | 156 | 100 (17/17) | 0 (0/6) | 0 (0/17) | 100 (6/6) | 9.91E-06 | 0.008519322 | 2.15E-05 | type II restriction-modification system regulatory protein |
| group_990 | 738 | 100 (17/17) | 0 (0/6) | 0 (0/17) | 100 (6/6) | 9.91E-06 | 0.008519322 | 2.15E-05 | multidrug ABC transporter permease |
| group_997 | 216 | 100 (17/17) | 0 (0/6) | 0 (0/17) | 100 (6/6) | 9.91E-06 | 0.008519322 | 2.15E-05 | hypothetical protein |
| group_996 | 1680 | 100 (17/17) | 0 (0/6) | 0 (0/17) | 100 (6/6) | 9.91E-06 | 0.008519322 | 2.15E-05 | FucA |
| group_995 | 1623 | 100 (17/17) | 0 (0/6) | 0 (0/17) | 100 (6/6) | 9.91E-06 | 0.008519322 | 2.15E-05 | exported glycosyl hydrolase |
| group_994 | 237 | 100 (17/17) | 0 (0/6) | 0 (0/17) | 100 (6/6) | 9.91E-06 | 0.008519322 | 2.15E-05 | hypothetical cytosolic protein |
| group_699 | 1011 | 0 (0/17) | 100 (6/6) | 100 (17/17) | 0 (0/6) | 9.91E-06 | 0.008519322 | 2.15E-05 | membrane protein |
| group_999 | 378 | 100 (17/17) | 0 (0/6) | 0 (0/17) | 100 (6/6) | 9.91E-06 | 0.008519322 | 2.15E-05 | hypothetical protein |
| group_998 | 411 | 100 (17/17) | 0 (0/6) | 0 (0/17) | 100 (6/6) | 9.91E-06 | 0.008519322 | 2.15E-05 | hypothetical protein |
| bglA | 297 | 0 (0/17) | 100 (6/6) | 100 (17/17) | 0 (0/6) | 9.91E-06 | 0.008519322 | 2.15E-05 | 6-phospho-beta-glucosidase AscB |
| sglT | 1542 | 100 (17/17) | 0 (0/6) | 0 (0/17) | 100 (6/6) | 9.91E-06 | 0.008519322 | 2.15E-05 | sodium:solute symporter family protein |
| group_1009 | 267 | 100 (17/17) | 0 (0/6) | 0 (0/17) | 100 (6/6) | 9.91E-06 | 0.008519322 | 2.15E-05 | phage protein |
| efeN_2 | 237 | 0 (0/17) | 100 (6/6) | 100 (17/17) | 0 (0/6) | 9.91E-06 | 0.008519322 | 2.15E-05 | peroxidase |
| efeN_1 | 630 | 0 (0/17) | 100 (6/6) | 100 (17/17) | 0 (0/6) | 9.91E-06 | 0.008519322 | 2.15E-05 | peroxidase |
| group_1008 | 153 | 100 (17/17) | 0 (0/6) | 0 (0/17) | 100 (6/6) | 9.91E-06 | 0.008519322 | 2.15E-05 | hypothetical protein |
| group_979 | 183 | 100 (17/17) | 0 (0/6) | 0 (0/17) | 100 (6/6) | 9.91E-06 | 0.008519322 | 2.15E-05 | hypothetical protein |
| group_978 | 141 | 100 (17/17) | 0 (0/6) | 0 (0/17) | 100 (6/6) | 9.91E-06 | 0.008519322 | 2.15E-05 | hypothetical protein |
| group_872 | 450 | 100 (17/17) | 0 (0/6) | 0 (0/17) | 100 (6/6) | 9.91E-06 | 0.008519322 | 2.15E-05 | hypothetical protein |
| group_873 | 456 | 100 (17/17) | 0 (0/6) | 0 (0/17) | 100 (6/6) | 9.91E-06 | 0.008519322 | 2.15E-05 | diadenosine tetraphosphate |
| group_759 | 270 | 0 (0/17) | 100 (6/6) | 100 (17/17) | 0 (0/6) | 9.91E-06 | 0.008519322 | 2.15E-05 | membrane protein |
| group_875 | 1578 | 100 (17/17) | 0 (0/6) | 0 (0/17) | 100 (6/6) | 9.91E-06 | 0.008519322 | 2.15E-05 | ABC transporter substrate-binding protein |
| group_876 | 1440 | 100 (17/17) | 0 (0/6) | 0 (0/17) | 100 (6/6) | 9.91E-06 | 0.008519322 | 2.15E-05 | hypothetical protein |
| group_755 | 813 | 0 (0/17) | 100 (6/6) | 100 (17/17) | 0 (0/6) | 9.91E-06 | 0.008519322 | 2.15E-05 | phage protein |
| group_970 | 1275 | 100 (17/17) | 0 (0/6) | 0 (0/17) | 100 (6/6) | 9.91E-06 | 0.008519322 | 2.15E-05 | Lantibiotic biosynthesis protein |
| group_973 | 612 | 100 (17/17) | 0 (0/6) | 0 (0/17) | 100 (6/6) | 9.91E-06 | 0.008519322 | 2.15E-05 | MutT/nudix family protein |
| group_972 | 345 | 100 (17/17) | 0 (0/6) | 0 (0/17) | 100 (6/6) | 9.91E-06 | 0.008519322 | 2.15E-05 | membrane protein |
| group_751 | 369 | 0 (0/17) | 100 (6/6) | 100 (17/17) | 0 (0/6) | 9.91E-06 | 0.008519322 | 2.15E-05 | bacteriophage repressor protein |
| group_974 | 189 | 100 (17/17) | 0 (0/6) | 0 (0/17) | 100 (6/6) | 9.91E-06 | 0.008519322 | 2.15E-05 | hypothetical protein |
| group_752 | 234 | 0 (0/17) | 100 (6/6) | 100 (17/17) | 0 (0/6) | 9.91E-06 | 0.008519322 | 2.15E-05 | hypothetical protein |
| ptrB | 1941 | 100 (17/17) | 0 (0/6) | 0 (0/17) | 100 (6/6) | 9.91E-06 | 0.008519322 | 2.15E-05 | prolyl oligopeptidase family protein |
| group_1041 | 246 | 100 (17/17) | 0 (0/6) | 0 (0/17) | 100 (6/6) | 9.91E-06 | 0.008519322 | 2.15E-05 | Uncharacterized protein conserved in bacteria |
| group_1043 | 207 | 100 (17/17) | 0 (0/6) | 0 (0/17) | 100 (6/6) | 9.91E-06 | 0.008519322 | 2.15E-05 | transposase |
| group_1045 | 165 | 100 (17/17) | 0 (0/6) | 0 (0/17) | 100 (6/6) | 9.91E-06 | 0.008519322 | 2.15E-05 | hypothetical protein |
| group_1044 | 240 | 100 (17/17) | 0 (0/6) | 0 (0/17) | 100 (6/6) | 9.91E-06 | 0.008519322 | 2.15E-05 | hypothetical protein |
| group_1046 | 477 | 100 (17/17) | 0 (0/6) | 0 (0/17) | 100 (6/6) | 9.91E-06 | 0.008519322 | 2.15E-05 | putative sortase-sorted surface anchored protein (pseudogene) |
| pblB | 3222 | 0 (0/17) | 100 (6/6) | 100 (17/17) | 0 (0/6) | 9.91E-06 | 0.008519322 | 2.15E-05 | bacteriophage PblB |
| leuB_2 | 156 | 100 (17/17) | 0 (0/6) | 0 (0/17) | 100 (6/6) | 9.91E-06 | 0.008519322 | 2.15E-05 | 3-isopropylmalate dehydrogenase |
| group_908 | 243 | 100 (17/17) | 0 (0/6) | 0 (0/17) | 100 (6/6) | 9.91E-06 | 0.008519322 | 2.15E-05 | putative DNA alkylation repair enzyme |
| group_909 | 957 | 100 (17/17) | 0 (0/6) | 0 (0/17) | 100 (6/6) | 9.91E-06 | 0.008519322 | 2.15E-05 | choline binding protein J |
| group_907 | 270 | 100 (17/17) | 0 (0/6) | 0 (0/17) | 100 (6/6) | 9.91E-06 | 0.008519322 | 2.15E-05 | abortive infection protein |
| group_900 | 270 | 100 (17/17) | 0 (0/6) | 0 (0/17) | 100 (6/6) | 9.91E-06 | 0.008519322 | 2.15E-05 | membrane protein |
| group_901 | 327 | 100 (17/17) | 0 (0/6) | 0 (0/17) | 100 (6/6) | 9.91E-06 | 0.008519322 | 2.15E-05 | BlpT protein, fusion |
| group_902 | 336 | 100 (17/17) | 0 (0/6) | 0 (0/17) | 100 (6/6) | 9.91E-06 | 0.008519322 | 2.15E-05 | hypothetical protein |
| group_903 | 255 | 100 (17/17) | 0 (0/6) | 0 (0/17) | 100 (6/6) | 9.91E-06 | 0.008519322 | 2.15E-05 | Prevent host death family protein |
| group_799 | 1095 | 0 (0/17) | 100 (6/6) | 100 (17/17) | 0 (0/6) | 9.91E-06 | 0.008519322 | 2.15E-05 | hypothetical protein |
| group_798 | 531 | 0 (0/17) | 100 (6/6) | 100 (17/17) | 0 (0/6) | 9.91E-06 | 0.008519322 | 2.15E-05 | hypothetical protein |
| group_271 | 615 | 0 (0/17) | 100 (6/6) | 100 (17/17) | 0 (0/6) | 9.91E-06 | 0.008519322 | 2.15E-05 | choline binding protein |
| group_377 | 819 | 0 (0/17) | 100 (6/6) | 100 (17/17) | 0 (0/6) | 9.91E-06 | 0.008519322 | 2.15E-05 | hypothetical protein |
| group_372 | 1047 | 0 (0/17) | 100 (6/6) | 100 (17/17) | 0 (0/6) | 9.91E-06 | 0.008519322 | 2.15E-05 | relaxase |
| group_358 | 1098 | 0 (0/17) | 100 (6/6) | 100 (17/17) | 0 (0/6) | 9.91E-06 | 0.008519322 | 2.15E-05 | histidine kinase |
| hsdM2 | 279 | 0 (0/17) | 100 (6/6) | 100 (17/17) | 0 (0/6) | 9.91E-06 | 0.008519322 | 2.15E-05 | type I restriction-modification system methylation subunit |
| zmpD | 5418 | 0 (0/17) | 100 (6/6) | 100 (17/17) | 0 (0/6) | 9.91E-06 | 0.008519322 | 2.15E-05 | zinc metalloprotease |
| group_874 | 1461 | 100 (17/17) | 0 (0/6) | 0 (0/17) | 100 (6/6) | 9.91E-06 | 0.008519322 | 2.15E-05 | dihydrolipoamide dehydrogenase |
| rmlD | 456 | 100 (17/17) | 0 (0/6) | 0 (0/17) | 100 (6/6) | 9.91E-06 | 0.008519322 | 2.15E-05 | dTDP-4-keto-L-rhamnose reductase |
| zmpB | 5688 | 100 (17/17) | 0 (0/6) | 0 (0/17) | 100 (6/6) | 9.91E-06 | 0.008519322 | 2.15E-05 | zinc metalloprotease ZmpB |
| moeB | 771 | 100 (17/17) | 0 (0/6) | 0 (0/17) | 100 (6/6) | 9.91E-06 | 0.008519322 | 2.15E-05 | HesA/MoeB/ThiF family protein |
| Int-Tn | 1164 | 100 (17/17) | 0 (0/6) | 0 (0/17) | 100 (6/6) | 9.91E-06 | 0.008519322 | 2.15E-05 | Integrase |
| group_476 | 930 | 0 (0/17) | 100 (6/6) | 100 (17/17) | 0 (0/6) | 9.91E-06 | 0.008519322 | 2.15E-05 | hypothetical protein |
| hlyB_2 | 774 | 100 (17/17) | 0 (0/6) | 0 (0/17) | 100 (6/6) | 9.91E-06 | 0.008519322 | 2.15E-05 | lantibiotic export protein |
| group_575 | 762 | 0 (0/17) | 100 (6/6) | 100 (17/17) | 0 (0/6) | 9.91E-06 | 0.008519322 | 2.15E-05 | DNA or RNA helicases of superfamily II |
| group_672 | 648 | 0 (0/17) | 100 (6/6) | 100 (17/17) | 0 (0/6) | 9.91E-06 | 0.008519322 | 2.15E-05 | Nicotinamide mononucleotide transporter |
| group_671 | 186 | 0 (0/17) | 100 (6/6) | 100 (17/17) | 0 (0/6) | 9.91E-06 | 0.008519322 | 2.15E-05 | hypothetical protein |
| dnaC_2 | 771 | 0 (0/17) | 100 (6/6) | 100 (17/17) | 0 (0/6) | 9.91E-06 | 0.008519322 | 2.15E-05 | DNA replication protein |
| blpO_2 | 231 | 100 (17/17) | 0 (0/6) | 0 (0/17) | 100 (6/6) | 9.91E-06 | 0.008519322 | 2.15E-05 | bacteriocin BlpO |
| rpiR | 810 | 100 (17/17) | 0 (0/6) | 0 (0/17) | 100 (6/6) | 9.91E-06 | 0.008519322 | 2.15E-05 | transcriptional regulator |
| afr_2 | 1110 | 100 (17/17) | 0 (0/6) | 0 (0/17) | 100 (6/6) | 9.91E-06 | 0.008519322 | 2.15E-05 | Gfo/Idh/MocA family oxidoreductase |
| tcyA_2 | 831 | 100 (17/17) | 0 (0/6) | 0 (0/17) | 100 (6/6) | 9.91E-06 | 0.008519322 | 2.15E-05 | ABC transporter substrate-binding lipoprotein |
| ABC-MSP-truncation | 237 | 100 (17/17) | 0 (0/6) | 0 (0/17) | 100 (6/6) | 9.91E-06 | 0.008519322 | 2.15E-05 | ABC transporter |
| group_1949 | 138 | 100 (17/17) | 0 (0/6) | 0 (0/17) | 100 (6/6) | 9.91E-06 | 0.008519322 | 2.15E-05 | hypothetical protein |
| dcm1 | 1317 | 0 (0/17) | 100 (6/6) | 100 (17/17) | 0 (0/6) | 9.91E-06 | 0.008519322 | 2.15E-05 | DNA (cytosine-5-)-methyltransferase |
| group_934 | 270 | 100 (17/17) | 0 (0/6) | 0 (0/17) | 100 (6/6) | 9.91E-06 | 0.008519322 | 2.15E-05 | bacteriocin BlpJ |
| group_931 | 624 | 100 (17/17) | 0 (0/6) | 0 (0/17) | 100 (6/6) | 9.91E-06 | 0.008519322 | 2.15E-05 | Transporter |
| group_930 | 243 | 100 (17/17) | 0 (0/6) | 0 (0/17) | 100 (6/6) | 9.91E-06 | 0.008519322 | 2.15E-05 | MFS transporter |
| group_932 | 150 | 100 (17/17) | 0 (0/6) | 0 (0/17) | 100 (6/6) | 9.91E-06 | 0.008519322 | 2.15E-05 | hypothetical protein |
| group_938 | 252 | 100 (17/17) | 0 (0/6) | 0 (0/17) | 100 (6/6) | 9.91E-06 | 0.008519322 | 2.15E-05 | transcriptional regulator |
| group_1005 | 216 | 100 (17/17) | 0 (0/6) | 0 (0/17) | 100 (6/6) | 9.91E-06 | 0.008519322 | 2.15E-05 | hypothetical protein |
| group_1007 | 297 | 100 (17/17) | 0 (0/6) | 0 (0/17) | 100 (6/6) | 9.91E-06 | 0.008519322 | 2.15E-05 | phage protein |
| group_1006 | 201 | 100 (17/17) | 0 (0/6) | 0 (0/17) | 100 (6/6) | 9.91E-06 | 0.008519322 | 2.15E-05 | hypothetical protein |
| group_1001 | 606 | 100 (17/17) | 0 (0/6) | 0 (0/17) | 100 (6/6) | 9.91E-06 | 0.008519322 | 2.15E-05 | hypothetical protein |
| group_1000 | 429 | 100 (17/17) | 0 (0/6) | 0 (0/17) | 100 (6/6) | 9.91E-06 | 0.008519322 | 2.15E-05 | phage protein |
| group_1003 | 897 | 100 (17/17) | 0 (0/6) | 0 (0/17) | 100 (6/6) | 9.91E-06 | 0.008519322 | 2.15E-05 | GTP-binding protein |
| group_1002 | 174 | 100 (17/17) | 0 (0/6) | 0 (0/17) | 100 (6/6) | 9.91E-06 | 0.008519322 | 2.15E-05 | hypothetical protein |
| dhaM_2 | 159 | 0 (0/17) | 100 (6/6) | 100 (17/17) | 0 (0/6) | 9.91E-06 | 0.008519322 | 2.15E-05 | PTS system fructose family transporter subunit IIA |
| group_967 | 324 | 100 (17/17) | 0 (0/6) | 0 (0/17) | 100 (6/6) | 9.91E-06 | 0.008519322 | 2.15E-05 | branched-chain amino acid transport protein |
| group_339 | 459 | 0 (0/17) | 100 (6/6) | 100 (17/17) | 0 (0/6) | 9.91E-06 | 0.008519322 | 2.15E-05 | MmcQ family protein |
| group_702 | 180 | 0 (0/17) | 100 (6/6) | 100 (17/17) | 0 (0/6) | 9.91E-06 | 0.008519322 | 2.15E-05 | DNA-binding protein |
| group_703 | 957 | 0 (0/17) | 100 (6/6) | 100 (17/17) | 0 (0/6) | 9.91E-06 | 0.008519322 | 2.15E-05 | autolysin |
| group_700 | 234 | 0 (0/17) | 100 (6/6) | 100 (17/17) | 0 (0/6) | 9.91E-06 | 0.008519322 | 2.15E-05 | adenylosuccinate lyase |
| group_706 | 351 | 0 (0/17) | 100 (6/6) | 100 (17/17) | 0 (0/6) | 9.91E-06 | 0.008519322 | 2.15E-05 | hypothetical protein |
| lagD_2 | 1317 | 100 (17/17) | 0 (0/6) | 0 (0/17) | 100 (6/6) | 9.91E-06 | 0.008519322 | 2.15E-05 | lantibiotic export protein |
| group_704 | 336 | 0 (0/17) | 100 (6/6) | 100 (17/17) | 0 (0/6) | 9.91E-06 | 0.008519322 | 2.15E-05 | phage holin, LL-H family |
| group_705 | 417 | 0 (0/17) | 100 (6/6) | 100 (17/17) | 0 (0/6) | 9.91E-06 | 0.008519322 | 2.15E-05 | putative holin 1 |
| group_708 | 3171 | 0 (0/17) | 100 (6/6) | 100 (17/17) | 0 (0/6) | 9.91E-06 | 0.008519322 | 2.15E-05 | PblB |
| lysA_2 | 1107 | 100 (17/17) | 0 (0/6) | 0 (0/17) | 100 (6/6) | 9.91E-06 | 0.008519322 | 2.15E-05 | Pyridoxal dependent decarboxylase |
| group_797 | 702 | 0 (0/17) | 100 (6/6) | 100 (17/17) | 0 (0/6) | 9.91E-06 | 0.008519322 | 2.15E-05 | hypothetical protein |
| group_836 | 204 | 0 (0/17) | 100 (6/6) | 100 (17/17) | 0 (0/6) | 9.91E-06 | 0.008519322 | 2.15E-05 | glucokinase |
| group_385 | 1782 | 0 (0/17) | 100 (6/6) | 100 (17/17) | 0 (0/6) | 9.91E-06 | 0.008519322 | 2.15E-05 | SigA binding protein |
| group_830 | 1410 | 0 (0/17) | 100 (6/6) | 100 (17/17) | 0 (0/6) | 9.91E-06 | 0.008519322 | 2.15E-05 | hypothetical protein |
| drrA | 729 | 100 (17/17) | 0 (0/6) | 0 (0/17) | 100 (6/6) | 9.91E-06 | 0.008519322 | 2.15E-05 | lantibiotic transport ATP-binding protein |
| group_707 | 204 | 0 (0/17) | 100 (6/6) | 100 (17/17) | 0 (0/6) | 9.91E-06 | 0.008519322 | 2.15E-05 | hypothetical protein |
| lagD_1 | 339 | 100 (17/17) | 0 (0/6) | 0 (0/17) | 100 (6/6) | 9.91E-06 | 0.008519322 | 2.15E-05 | transporter truncation |
| group_695 | 2109 | 0 (0/17) | 100 (6/6) | 100 (17/17) | 0 (0/6) | 9.91E-06 | 0.008519322 | 2.15E-05 | bacteriocin immunity protein |
| group_696 | 285 | 0 (0/17) | 100 (6/6) | 100 (17/17) | 0 (0/6) | 9.91E-06 | 0.008519322 | 2.15E-05 | bacteriocin |
| group_207 | 405 | 0 (0/17) | 100 (6/6) | 100 (17/17) | 0 (0/6) | 9.91E-06 | 0.008519322 | 2.15E-05 | transposase |
| nanA_3 | 918 | 100 (17/17) | 0 (0/6) | 0 (0/17) | 100 (6/6) | 9.91E-06 | 0.008519322 | 2.15E-05 | N-acetylneuraminate lyase |
| group_205 | 267 | 0 (0/17) | 100 (6/6) | 100 (17/17) | 0 (0/6) | 9.91E-06 | 0.008519322 | 2.15E-05 | transposase |
| group_636 | 456 | 0 (0/17) | 100 (6/6) | 100 (17/17) | 0 (0/6) | 9.91E-06 | 0.008519322 | 2.15E-05 | MutT/NUDIX family protein |
| group_637 | 351 | 0 (0/17) | 100 (6/6) | 100 (17/17) | 0 (0/6) | 9.91E-06 | 0.008519322 | 2.15E-05 | transposase, ISSmi4 |
| group_634 | 1479 | 0 (0/17) | 100 (6/6) | 100 (17/17) | 0 (0/6) | 9.91E-06 | 0.008519322 | 2.15E-05 | sodium-dependent transporter |
| group_632 | 153 | 0 (0/17) | 100 (6/6) | 100 (17/17) | 0 (0/6) | 9.91E-06 | 0.008519322 | 2.15E-05 | ABC transporter substrate binding protein |
| group_639 | 561 | 0 (0/17) | 100 (6/6) | 100 (17/17) | 0 (0/6) | 9.91E-06 | 0.008519322 | 2.15E-05 | acetyltransferase, gnat family |
| group_971 | 339 | 100 (17/17) | 0 (0/6) | 0 (0/17) | 100 (6/6) | 9.91E-06 | 0.008519322 | 2.15E-05 | antibiotic biosynthesis monooxygenase subfamily protein |
| leuA_2 | 420 | 0 (0/17) | 100 (6/6) | 100 (17/17) | 0 (0/6) | 9.91E-06 | 0.008519322 | 2.15E-05 | 2-isopropylmalate synthase |
| group_754 | 159 | 0 (0/17) | 100 (6/6) | 100 (17/17) | 0 (0/6) | 9.91E-06 | 0.008519322 | 2.15E-05 | hypothetical protein |
| group_756 | 1149 | 0 (0/17) | 100 (6/6) | 100 (17/17) | 0 (0/6) | 9.91E-06 | 0.008519322 | 2.15E-05 | integrase |
| group_750 | 147 | 0 (0/17) | 100 (6/6) | 100 (17/17) | 0 (0/6) | 9.91E-06 | 0.008519322 | 2.15E-05 | hypothetical protein |
| dnaD_2 | 810 | 100 (17/17) | 0 (0/6) | 0 (0/17) | 100 (6/6) | 9.91E-06 | 0.008519322 | 2.15E-05 | Phage replication protein |
| group_976 | 756 | 100 (17/17) | 0 (0/6) | 0 (0/17) | 100 (6/6) | 9.91E-06 | 0.008519322 | 2.15E-05 | Zn-finger |
| group_448 | 405 | 0 (0/17) | 100 (6/6) | 100 (17/17) | 0 (0/6) | 9.91E-06 | 0.008519322 | 2.15E-05 | dTDP-4-keto-L-rhamnose reductase |
| group_44 | 1275 | 0 (0/17) | 100 (6/6) | 100 (17/17) | 0 (0/6) | 9.91E-06 | 0.008519322 | 2.15E-05 | transposase |
| group_113 | 294 | 0 (0/17) | 100 (6/6) | 100 (17/17) | 0 (0/6) | 9.91E-06 | 0.008519322 | 2.15E-05 | transposase-like protein, IS1381 ISSpn7 |
| group_940 | 315 | 100 (17/17) | 0 (0/6) | 0 (0/17) | 100 (6/6) | 9.91E-06 | 0.008519322 | 2.15E-05 | hypothetical protein |
| group_942 | 123 | 100 (17/17) | 0 (0/6) | 0 (0/17) | 100 (6/6) | 9.91E-06 | 0.008519322 | 2.15E-05 | hypothetical protein |
| group_767 | 837 | 0 (0/17) | 100 (6/6) | 100 (17/17) | 0 (0/6) | 9.91E-06 | 0.008519322 | 2.15E-05 | abortive infection protein AbiGII |
| pclA | 6849 | 100 (17/17) | 0 (0/6) | 0 (0/17) | 100 (6/6) | 9.91E-06 | 0.008519322 | 2.15E-05 | putative collagen-like surface-anchored protein |
| group_984 | 393 | 100 (17/17) | 0 (0/6) | 0 (0/17) | 100 (6/6) | 9.91E-06 | 0.008519322 | 2.15E-05 | hypothetical protein |
| group_985 | 300 | 100 (17/17) | 0 (0/6) | 0 (0/17) | 100 (6/6) | 9.91E-06 | 0.008519322 | 2.15E-05 | hypothetical protein |
| group_986 | 384 | 100 (17/17) | 0 (0/6) | 0 (0/17) | 100 (6/6) | 9.91E-06 | 0.008519322 | 2.15E-05 | hypothetical protein |
| group_987 | 192 | 100 (17/17) | 0 (0/6) | 0 (0/17) | 100 (6/6) | 9.91E-06 | 0.008519322 | 2.15E-05 | alanine aminotransferase |
| group_982 | 324 | 100 (17/17) | 0 (0/6) | 0 (0/17) | 100 (6/6) | 9.91E-06 | 0.008519322 | 2.15E-05 | transposase, ISSmi4 |
| group_983 | 666 | 100 (17/17) | 0 (0/6) | 0 (0/17) | 100 (6/6) | 9.91E-06 | 0.008519322 | 2.15E-05 | hypothetical protein |
| group_988 | 195 | 100 (17/17) | 0 (0/6) | 0 (0/17) | 100 (6/6) | 9.91E-06 | 0.008519322 | 2.15E-05 | hypothetical protein |
| group_489 | 234 | 0 (0/17) | 100 (6/6) | 100 (17/17) | 0 (0/6) | 9.91E-06 | 0.008519322 | 2.15E-05 | chlorohydrolase |
| group_805 | 195 | 0 (0/17) | 100 (6/6) | 100 (17/17) | 0 (0/6) | 9.91E-06 | 0.008519322 | 2.15E-05 | transposase-like protein, IS1381 ISSpn7 |
| group_804 | 753 | 0 (0/17) | 100 (6/6) | 100 (17/17) | 0 (0/6) | 9.91E-06 | 0.008519322 | 2.15E-05 | ABC transporter permease |
| group_807 | 198 | 0 (0/17) | 100 (6/6) | 100 (17/17) | 0 (0/6) | 9.91E-06 | 0.008519322 | 2.15E-05 | helix-turn-helix domain-containing protein |
| group_969 | 372 | 100 (17/17) | 0 (0/6) | 0 (0/17) | 100 (6/6) | 9.91E-06 | 0.008519322 | 2.15E-05 | hypothetical protein |
| group_801 | 1029 | 0 (0/17) | 100 (6/6) | 100 (17/17) | 0 (0/6) | 9.91E-06 | 0.008519322 | 2.15E-05 | UDP-glucose 4-epimerase |
| group_800 | 672 | 0 (0/17) | 100 (6/6) | 100 (17/17) | 0 (0/6) | 9.91E-06 | 0.008519322 | 2.15E-05 | hypothetical protein |
| group_803 | 408 | 0 (0/17) | 100 (6/6) | 100 (17/17) | 0 (0/6) | 9.91E-06 | 0.008519322 | 2.15E-05 | major facilitator superfamily permease |
| group_749 | 375 | 0 (0/17) | 100 (6/6) | 100 (17/17) | 0 (0/6) | 9.91E-06 | 0.008519322 | 2.15E-05 | hypothetical protein |
| group_746 | 162 | 0 (0/17) | 100 (6/6) | 100 (17/17) | 0 (0/6) | 9.91E-06 | 0.008519322 | 2.15E-05 | hypothetical protein |
| group_744 | 714 | 0 (0/17) | 100 (6/6) | 100 (17/17) | 0 (0/6) | 9.91E-06 | 0.008519322 | 2.15E-05 | gp15 |
| group_961 | 471 | 100 (17/17) | 0 (0/6) | 0 (0/17) | 100 (6/6) | 9.91E-06 | 0.008519322 | 2.15E-05 | HIT family protein |
| group_743 | 258 | 0 (0/17) | 100 (6/6) | 100 (17/17) | 0 (0/6) | 9.91E-06 | 0.008519322 | 2.15E-05 | phage protein |
| group_740 | 858 | 0 (0/17) | 100 (6/6) | 100 (17/17) | 0 (0/6) | 9.91E-06 | 0.008519322 | 2.15E-05 | gp19 |
| group_741 | 297 | 0 (0/17) | 100 (6/6) | 100 (17/17) | 0 (0/6) | 9.91E-06 | 0.008519322 | 2.15E-05 | gp18 |
| ycjP_1 | 930 | 100 (17/17) | 0 (0/6) | 0 (0/17) | 100 (6/6) | 9.91E-06 | 0.008519322 | 2.15E-05 | protein LplC |
| group_833 | 333 | 0 (0/17) | 100 (6/6) | 100 (17/17) | 0 (0/6) | 9.91E-06 | 0.008519322 | 2.15E-05 | membrane protein |
| group_249 | 744 | 0 (0/17) | 100 (6/6) | 100 (17/17) | 0 (0/6) | 9.91E-06 | 0.008519322 | 2.15E-05 | topology modulation protein |
| rsmE | 744 | 100 (17/17) | 0 (0/6) | 0 (0/17) | 100 (6/6) | 9.91E-06 | 0.008519322 | 2.15E-05 | 16S ribosomal RNA methyltransferase RsmE |
| group_361 | 228 | 0 (0/17) | 100 (6/6) | 100 (17/17) | 0 (0/6) | 9.91E-06 | 0.008519322 | 2.15E-05 | immunity protein |
| group_367 | 849 | 0 (0/17) | 100 (6/6) | 100 (17/17) | 0 (0/6) | 9.91E-06 | 0.008519322 | 2.15E-05 | resolvase family site-specific recombinase |
| group_369 | 327 | 100 (17/17) | 0 (0/6) | 0 (0/17) | 100 (6/6) | 9.91E-06 | 0.008519322 | 2.15E-05 | relaxase/mobilization nuclease domain |
| gutB | 1044 | 0 (0/17) | 100 (6/6) | 100 (17/17) | 0 (0/6) | 9.91E-06 | 0.008519322 | 2.15E-05 | L-iditol 2-dehydrogenase |
| yheI_2 | 1593 | 100 (17/17) | 0 (0/6) | 0 (0/17) | 100 (6/6) | 9.91E-06 | 0.008519322 | 2.15E-05 | peptide ABC transporter ATP-binding protein/permease |
| group_959 | 297 | 100 (17/17) | 0 (0/6) | 0 (0/17) | 100 (6/6) | 9.91E-06 | 0.008519322 | 2.15E-05 | bacteriocin |
| rgg_2 | 342 | 100 (17/17) | 0 (0/6) | 0 (0/17) | 100 (6/6) | 9.91E-06 | 0.008519322 | 2.15E-05 | transcriptional activator, Rgg/GadR/MutR family protein |
| gspA_3 | 2445 | 100 (17/17) | 0 (0/6) | 0 (0/17) | 100 (6/6) | 9.91E-06 | 0.008519322 | 2.15E-05 | putative glycosyltransferase |
| group_563 | 132 | 0 (0/17) | 100 (6/6) | 100 (17/17) | 0 (0/6) | 9.91E-06 | 0.008519322 | 2.15E-05 | IS1381, transposase OrfA |
| group_649 | 381 | 0 (0/17) | 100 (6/6) | 100 (17/17) | 0 (0/6) | 9.91E-06 | 0.008519322 | 2.15E-05 | hypothetical protein |
| group_645 | 1464 | 0 (0/17) | 100 (6/6) | 100 (17/17) | 0 (0/6) | 9.91E-06 | 0.008519322 | 2.15E-05 | type I restriction-modification system M protein |
| group_644 | 255 | 0 (0/17) | 100 (6/6) | 100 (17/17) | 0 (0/6) | 9.91E-06 | 0.008519322 | 2.15E-05 | BlpM |
| group_643 | 162 | 0 (0/17) | 100 (6/6) | 100 (17/17) | 0 (0/6) | 9.91E-06 | 0.008519322 | 2.15E-05 | immunity protein |
| group_642 | 372 | 0 (0/17) | 100 (6/6) | 100 (17/17) | 0 (0/6) | 9.91E-06 | 0.008519322 | 2.15E-05 | immunity protein BlpL |
| desK | 1098 | 100 (17/17) | 0 (0/6) | 0 (0/17) | 100 (6/6) | 9.91E-06 | 0.008519322 | 2.15E-05 | histidine sensor kinase protein |
| PTS-EII_3 | 444 | 100 (17/17) | 0 (0/6) | 0 (0/17) | 100 (6/6) | 9.91E-06 | 0.008519322 | 2.15E-05 | PTS system IIA component |
| hpaIIM | 1359 | 0 (0/17) | 100 (6/6) | 100 (17/17) | 0 (0/6) | 9.91E-06 | 0.008519322 | 2.15E-05 | putative DNA methylase |
| ycjO_1 | 933 | 100 (17/17) | 0 (0/6) | 0 (0/17) | 100 (6/6) | 9.91E-06 | 0.008519322 | 2.15E-05 | protein LplB |
| artI | 702 | 100 (17/17) | 0 (0/6) | 0 (0/17) | 100 (6/6) | 9.91E-06 | 0.008519322 | 2.15E-05 | ABC transporter substrate binding lipoprotein-amino acid transport |
| ntpB | 1386 | 100 (17/17) | 0 (0/6) | 0 (0/17) | 100 (6/6) | 9.91E-06 | 0.008519322 | 2.15E-05 | V-type H+-ATPase, subunit B |
| ntpC | 1008 | 100 (17/17) | 0 (0/6) | 0 (0/17) | 100 (6/6) | 9.91E-06 | 0.008519322 | 2.15E-05 | V-type H+-ATPase, subunit C |
| epiD | 558 | 100 (17/17) | 0 (0/6) | 0 (0/17) | 100 (6/6) | 9.91E-06 | 0.008519322 | 2.15E-05 | flavoprotein |
| ntpD | 612 | 100 (17/17) | 0 (0/6) | 0 (0/17) | 100 (6/6) | 9.91E-06 | 0.008519322 | 2.15E-05 | ATP synthase, subunit D |
| ntpE | 582 | 100 (17/17) | 0 (0/6) | 0 (0/17) | 100 (6/6) | 9.91E-06 | 0.008519322 | 2.15E-05 | V-type H+-ATPase, subunit E |
| group_698 | 363 | 0 (0/17) | 100 (6/6) | 100 (17/17) | 0 (0/6) | 9.91E-06 | 0.008519322 | 2.15E-05 | chlorohydrolase |
| bltD_2 | 480 | 0 (0/17) | 100 (6/6) | 100 (17/17) | 0 (0/6) | 9.91E-06 | 0.008519322 | 2.15E-05 | acetyltransferase |
| blpI | 198 | 100 (17/17) | 0 (0/6) | 0 (0/17) | 100 (6/6) | 9.91E-06 | 0.008519322 | 2.15E-05 | bacteriocin BlpI |
| group_1950 | 138 | 100 (17/17) | 0 (0/6) | 0 (0/17) | 100 (6/6) | 9.91E-06 | 0.008519322 | 2.15E-05 | hypothetical protein |
| group_925 | 873 | 100 (17/17) | 0 (0/6) | 0 (0/17) | 100 (6/6) | 9.91E-06 | 0.008519322 | 2.15E-05 | transcriptional regulator |
| group_144 | 546 | 100 (17/17) | 0 (0/6) | 0 (0/17) | 100 (6/6) | 9.91E-06 | 0.008519322 | 2.15E-05 | IS1380-Spn1 transposase |
| group_1031 | 2295 | 100 (17/17) | 0 (0/6) | 0 (0/17) | 100 (6/6) | 9.91E-06 | 0.008519322 | 2.15E-05 | large secreted protein |
| group_1036 | 327 | 100 (17/17) | 0 (0/6) | 0 (0/17) | 100 (6/6) | 9.91E-06 | 0.008519322 | 2.15E-05 | competence protein ComGC |
| group_1037 | 477 | 100 (17/17) | 0 (0/6) | 0 (0/17) | 100 (6/6) | 9.91E-06 | 0.008519322 | 2.15E-05 | acetyltransferase |
| group_1038 | 183 | 100 (17/17) | 0 (0/6) | 0 (0/17) | 100 (6/6) | 9.91E-06 | 0.008519322 | 2.15E-05 | hypothetical protein |
| group_1039 | 210 | 100 (17/17) | 0 (0/6) | 0 (0/17) | 100 (6/6) | 9.91E-06 | 0.008519322 | 2.15E-05 | chlorohydrolase |
| gsiA | 1983 | 0 (0/17) | 100 (6/6) | 100 (17/17) | 0 (0/6) | 9.91E-06 | 0.008519322 | 2.15E-05 | ABC transporter ATP-binding protein |
| gsiB | 1629 | 0 (0/17) | 100 (6/6) | 100 (17/17) | 0 (0/6) | 9.91E-06 | 0.008519322 | 2.15E-05 | oligopeptide ABC transporteroligopeptide-binding protein |
| group_737 | 195 | 0 (0/17) | 100 (6/6) | 100 (17/17) | 0 (0/6) | 9.91E-06 | 0.008519322 | 2.15E-05 | hypothetical protein |
| group_736 | 228 | 0 (0/17) | 100 (6/6) | 100 (17/17) | 0 (0/6) | 9.91E-06 | 0.008519322 | 2.15E-05 | phage protein |
| group_735 | 168 | 0 (0/17) | 100 (6/6) | 100 (17/17) | 0 (0/6) | 9.91E-06 | 0.008519322 | 2.15E-05 | hypothetical protein |
| group_734 | 327 | 0 (0/17) | 100 (6/6) | 100 (17/17) | 0 (0/6) | 9.91E-06 | 0.008519322 | 2.15E-05 | gp24 |
| group_733 | 426 | 0 (0/17) | 100 (6/6) | 100 (17/17) | 0 (0/6) | 9.91E-06 | 0.008519322 | 2.15E-05 | phage protein |
| group_732 | 126 | 0 (0/17) | 100 (6/6) | 100 (17/17) | 0 (0/6) | 9.91E-06 | 0.008519322 | 2.15E-05 | DNA N-4 cytosine methyltransferase |
| group_731 | 354 | 0 (0/17) | 100 (6/6) | 100 (17/17) | 0 (0/6) | 9.91E-06 | 0.008519322 | 2.15E-05 | DNA N-4 cytosine methyltransferase |
| group_730 | 390 | 0 (0/17) | 100 (6/6) | 100 (17/17) | 0 (0/6) | 9.91E-06 | 0.008519322 | 2.15E-05 | phage protein |
| ABC-NDB | 741 | 100 (17/17) | 0 (0/6) | 0 (0/17) | 100 (6/6) | 9.91E-06 | 0.008519322 | 2.15E-05 | ABC transporter ATP-binding protein |
| appB | 951 | 0 (0/17) | 100 (6/6) | 100 (17/17) | 0 (0/6) | 9.91E-06 | 0.008519322 | 2.15E-05 | peptide ABC transporter permease |
| relE | 264 | 0 (0/17) | 100 (6/6) | 100 (17/17) | 0 (0/6) | 9.91E-06 | 0.008519322 | 2.15E-05 | plasmid stabilisation system protein |
| fliY | 738 | 0 (0/17) | 100 (6/6) | 100 (17/17) | 0 (0/6) | 9.91E-06 | 0.008519322 | 2.15E-05 | ABC transporter substrate binding lipoprotein-amino acid transport |
| group_218 | 414 | 0 (0/17) | 100 (6/6) | 100 (17/17) | 0 (0/6) | 9.91E-06 | 0.008519322 | 2.15E-05 | transposase |
| ntpK | 477 | 100 (17/17) | 0 (0/6) | 0 (0/17) | 100 (6/6) | 9.91E-06 | 0.008519322 | 2.15E-05 | V-type H+-ATPase, subunit K |
| pezA | 477 | 0 (0/17) | 100 (6/6) | 100 (17/17) | 0 (0/6) | 9.91E-06 | 0.008519322 | 2.15E-05 | transcriptional regulator |
| mta | 741 | 0 (0/17) | 100 (6/6) | 100 (17/17) | 0 (0/6) | 9.91E-06 | 0.008519322 | 2.15E-05 | MerR family transcriptional regulator |
| rlmN_2 | 300 | 100 (17/17) | 0 (0/6) | 0 (0/17) | 100 (6/6) | 9.91E-06 | 0.008519322 | 2.15E-05 | radical SAM enzyme, Cfr family |
| pezT | 762 | 0 (0/17) | 100 (6/6) | 100 (17/17) | 0 (0/6) | 9.91E-06 | 0.008519322 | 2.15E-05 | signal recognition particle GTPase |
| glnQ-truncation_2 | 345 | 100 (17/17) | 0 (0/6) | 0 (0/17) | 100 (6/6) | 9.91E-06 | 0.008519322 | 2.15E-05 | glutamine ABC transporter ATP-binding protein |
| group_638 | 756 | 0 (0/17) | 100 (6/6) | 100 (17/17) | 0 (0/6) | 9.91E-06 | 0.008519322 | 2.15E-05 | Zn-finger |
| ntpA | 1776 | 100 (17/17) | 0 (0/6) | 0 (0/17) | 100 (6/6) | 9.91E-06 | 0.008519322 | 2.15E-05 | V-type ATP synthase subunit A |
| group_822 | 780 | 0 (0/17) | 100 (6/6) | 100 (17/17) | 0 (0/6) | 9.91E-06 | 0.008519322 | 2.15E-05 | ribonuclease HII |
| Fucolectin-rel | 3018 | 0 (0/17) | 100 (6/6) | 100 (17/17) | 0 (0/6) | 9.91E-06 | 0.008519322 | 2.15E-05 | blood group cleaving endo-beta-galactosidase |
| ntpF | 321 | 100 (17/17) | 0 (0/6) | 0 (0/17) | 100 (6/6) | 9.91E-06 | 0.008519322 | 2.15E-05 | V-type H+-ATPase, subunit F |
| group_513 | 342 | 0 (0/17) | 100 (6/6) | 100 (17/17) | 0 (0/6) | 9.91E-06 | 0.008519322 | 2.15E-05 | hypothetical protein |
| group_57 | 834 | 100 (17/17) | 0 (0/6) | 0 (0/17) | 100 (6/6) | 9.91E-06 | 0.008519322 | 2.15E-05 | IS3-Spn1 transposase, truncation |
| group_806 | 1980 | 0 (0/17) | 100 (6/6) | 100 (17/17) | 0 (0/6) | 9.91E-06 | 0.008519322 | 2.15E-05 | DNA or RNA helicases of superfamily II |
| mutX_1 | 468 | 100 (17/17) | 0 (0/6) | 0 (0/17) | 100 (6/6) | 9.91E-06 | 0.008519322 | 2.15E-05 | MutT/nudix family protein |
| group_2566 | 342 | 100 (17/17) | 0 (0/6) | 0 (0/17) | 100 (6/6) | 9.91E-06 | 0.008519322 | 2.15E-05 | IS3-Spn1 transposase, truncation |

Panagiotou et al. 2020 Supplemental information

**Full-length Western Blots**

**Figure 2B** (left panel) Cytosol –Full-Length Western Blot


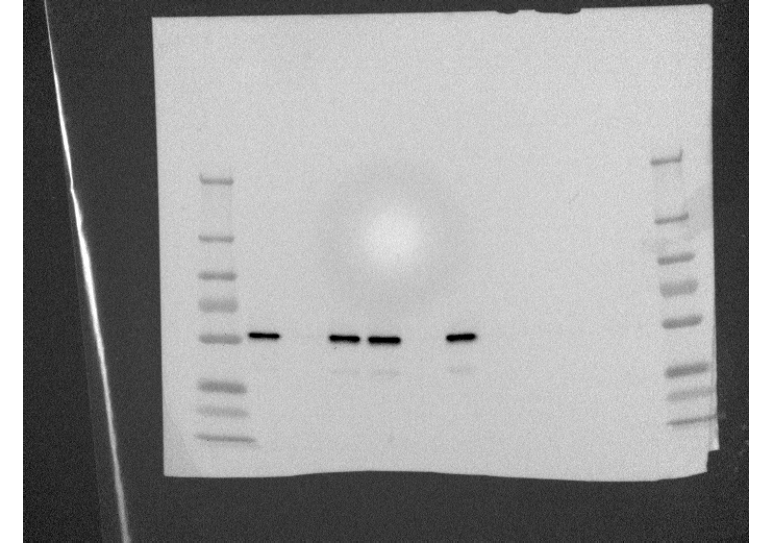


**Figure 2B** (right panel) Cell Wall –Full-Length Western Blot


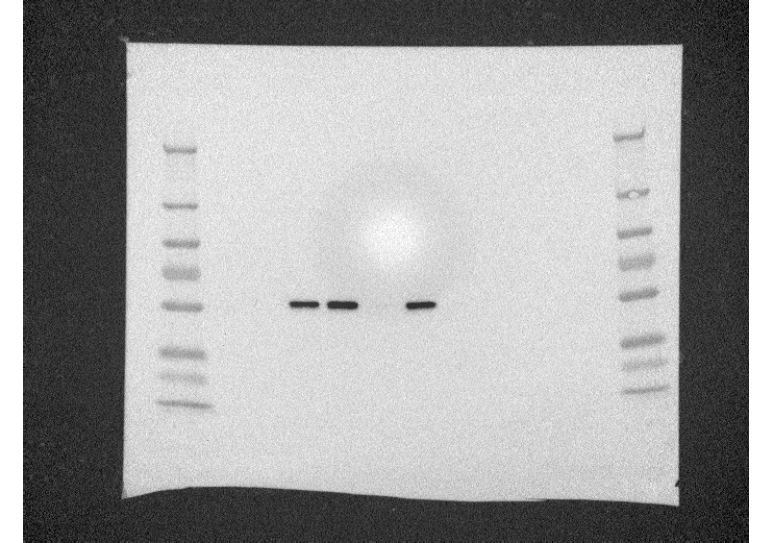

Supplement: Supplementary file 1 — Supplementary Information. [file 41598_2020_73454_MOESM1_ESM.docx]
